# A Combined Pathway and Regional Heritability Analysis Indicates NETRIN1 Pathway Is Associated With Major Depressive Disorder

**DOI:** 10.1016/j.biopsych.2016.04.017

**Published:** 2017-02-15

**Authors:** Yanni Zeng, Pau Navarro, Ana M. Fernandez-Pujals, Lynsey S. Hall, Toni-Kim Clarke, Pippa A. Thomson, Blair H. Smith, Lynne J. Hocking, Sandosh Padmanabhan, Caroline Hayward, Donald J. MacIntyre, Naomi R. Wray, Major Depressive Disorder Working Group of the Psychiatric Genomics Consortium, Ian J. Deary, David J. Porteous, Chris S. Haley, Andrew M. McIntosh

**Affiliations:** a Division of Psychiatry, University of Edinburgh, Edinburgh, United Kingdom; b MRC Human Genetics Unit, University of Edinburgh, Edinburgh, United Kingdom; c Centre for Cognitive Ageing and Cognitive Epidemiology, University of Edinburgh, Edinburgh, United Kingdom; d Medical Genetics Section, University of Edinburgh, Edinburgh, United Kingdom; e Generation Scotland, University of Edinburgh, Edinburgh, United Kingdom; f Centre for Genomic and Experimental Medicine, University of Edinburgh, Edinburgh, United Kingdom; g Institute of Genetics and Molecular Medicine, Department of Psychology, University of Edinburgh, Edinburgh, United Kingdom; h The Roslin Institute and Royal (Dick) School of Veterinary Sciences, University of Edinburgh, Edinburgh, United Kingdom; i Division of Population Health Sciences, University of Dundee, Dundee, United Kingdom; j Division of Applied Health Sciences, University of Aberdeen, Aberdeen, United Kingdom; k Institute of Cardiovascular and Medical Sciences, University of Glasgow, Glasgow, United Kingdom; l Queensland Brain Institute, University of Queensland, St Lucia, Queensland, Australia

**Keywords:** *DCC*, MDD, NETRIN1, Pathway analysis, Polygenic risk score, Regional heritability

## Abstract

**Background:**

Genome-wide association studies (GWASs) of major depressive disorder (MDD) have identified few significant associations. Testing the aggregation of genetic variants, in particular biological pathways, may be more powerful. Regional heritability analysis can be used to detect genomic regions that contribute to disease risk.

**Methods:**

We integrated pathway analysis and multilevel regional heritability analyses in a pipeline designed to identify MDD-associated pathways. The pipeline was applied to two independent GWAS samples [Generation Scotland: The Scottish Family Health Study (GS:SFHS, *N* = 6455) and Psychiatric Genomics Consortium (PGC:MDD) (*N* = 18,759)]. A polygenic risk score (PRS) composed of single nucleotide polymorphisms from the pathway most consistently associated with MDD was created, and its accuracy to predict MDD, using area under the curve, logistic regression, and linear mixed model analyses, was tested.

**Results:**

In GS:SFHS, four pathways were significantly associated with MDD, and two of these explained a significant amount of pathway-level regional heritability. In PGC:MDD, one pathway was significantly associated with MDD. Pathway-level regional heritability was significant in this pathway in one subset of PGC:MDD. For both samples the regional heritabilities were further localized to the gene and subregion levels. The NETRIN1 signaling pathway showed the most consistent association with MDD across the two samples. PRSs from this pathway showed competitive predictive accuracy compared with the whole-genome PRSs when using area under the curve statistics, logistic regression, and linear mixed model.

**Conclusions:**

These post-GWAS analyses highlight the value of combining multiple methods on multiple GWAS data for the identification of risk pathways for MDD. The NETRIN1 signaling pathway is identified as a candidate pathway for MDD and should be explored in further large population studies.

Major depressive disorder (MDD) contributes 8.2% of the global burden of disease (1). Twin studies have estimated the narrow sense heritability of MDD to be 37%, confirming the involvement of genetic factors in MDD (2). However, published genome-wide association studies (GWASs) of MDD have only detected two loci associated with recurrent MDD at genome-wide significance in a study of Chinese women (3, 4), despite the success of GWASs for other psychiatric disorders (5). Additional methods for detecting the aggregate effects of sub–genome-wide significant risk variants are required to better extract information from available data.

Two lessons relevant to MDD can be learned from previous studies of polygenic diseases. First, disease-associated variants are enriched in functionally annotated regions of the genome (6). Second, the small signals from individual genetic variants contrast with the stronger signals from individual pathways (7). With the use of GWAS summary statistics, a recent Psychiatric Genomics Consortium (PGC) study identified disease-specific and shared pathways across multiple psychiatric diseases (8). These findings suggest that the cumulative effects from single variants converge on biological pathways and that the pathways themselves may be more tractable targets for GWASs. In the present study, we sought to test whether the aggregate effects of low-penetrance variants become detectable at the pathway level in MDD.

Various approaches have been developed to identify the association between pathways and phenotype. Methods designed to be applied to raw genotypes or summary statistics of GWASs have been developed (9). The optimal method should depend on the data type available and the research goals. For instance, a pathway-based study demonstrated the feasibility of identifying pathways and genes associated with schizophrenia using analytic methods that are designed for different data types (raw genotypes and GWAS summary statistics) in three independent samples (10).

Genomic restricted maximum likelihood (GREML) analysis methods (11) can be used to estimate the additive variance contributed from all the genotyped single nucleotide polymorphisms (SNPs) by using linear mixed modeling (LMM). They may also be adapted to further partition the variance components by functionally annotated SNP categories (11, 12, 13). Generally, for polygenic traits the proportion of the phenotypic variance explained by the SNPs was proportional to the number of SNPs involved (14). However, genomic regions that explain more heritability than expected when accounting for the number of SNPs they contain have been uncovered. These regions usually overlap with regulatory, genic, or conserved regions of the human genome (12, 13, 15, 16). The locally enriched heritability is defined as regional heritability. Regional heritability analysis can be applied to identify genomic regions that contribute a significant proportion of heritability as an alternative association test (17). With the use of this method, a recent study identified more genomic regions that reached the suggestive level of significance than GWASs, suggesting that it is capable of capturing some of the signals not detected by a single SNP association test (18).

In this study, we sought to identify biological pathways associated with MDD by making use of well-annotated molecular pathway databases and two independent samples of European ancestry. Each sample was run through a pipeline in which a non–hypothesis-driven pathway analysis was applied to identify MDD-associated pathways and that was followed by multilevel regional heritability analyses to quantify and narrow the genetic contribution from the candidate pathways. With the use of this pipeline, we observed overlaps in the identified MDD candidate pathways, genes, and subregions between samples. Finally, to test for the predictive value of the pathway most consistently associated with MDD across samples, we compared the predictive accuracy of pathway-derived MDD polygenic risk score (PRS) to whole-genome–derived PRS.

## Methods and Materials

The Tayside Research Ethics Committee (reference 05/S1401/89) provided ethical approval for the study.

### Data Sets

#### Generation Scotland: The Scottish Family Health Study (GS:SFHS)

This study included 21,387 subjects (8772 men, 12,615 women; mean age, 47.2 years). Participants were recruited from the registers of collaborating general practices by their Community Health Index (19). A Structured Clinical Interview for DSM-IV was used for the diagnosis of MDD mood disorders (20) (Supplement). By the time we performed this study, 9863 individuals were genotyped using the Illumina (San Diego, CA) Human OmniExpressExome-8- v1.0 array (21). Details of genotyping are described in detail elsewhere (22). Quality control (QC) and imputation method are described in the Supplement. In total, 592,690 genotyped and 2,163,848 imputed autosomal SNPs passed QC criteria and were used in subsequent analyses. Because close relatives can bias the pathway analysis and SNP heritability estimation, the function “--grm-cutoff 0.025” in GCTA was used to remove one of each pair of individuals with estimated relatedness larger than 0.025 while maximizing the remaining sample size (11); 6455 subjects (1123 MDD case subjects; 5332 control subjects) remained in the analyses described below.

#### PGC:MDD

The PGC provided summary statistics from the GWAS mega-analysis of MDD from the discovery phase and individual genotypes from the nine primary cohorts in this data set. These data included 18,759 subjects of European ancestry (9240 MDD case subjects; 9519 control subjects) (4). Case subjects were required to have a diagnosis of DSM-IV lifetime MDD (Supplement). Summary statistics included GWAS *p* values and odds ratio information for 1,235,110 SNPs after imputation using CEU+TSI HapMap3 data as reference (410 phased haplotypes). We performed additional QC of these summary statistics with inclusion thresholds info score ≥0.8, and minor allele frequency ≥0.01, after which 1,074,100 SNPs remained and were used in pathway analysis and polygenic risk profiling.

Imputed genotype data from nine PGC:MDD cohorts were provided by PGC for the regional heritability analysis. Best-guess imputed genotypes from each cohort were accepted at the same level of QC as GS:SFHS. After removing one of each pair of close relatives (*t* ≥ 0.025), the remaining 17,845 subjects were used in the downstream analyses (see Supplemental Table S1 for sample information).

### A Pipeline for Identification of Pathways Associated With MDD

**Figure 1:**
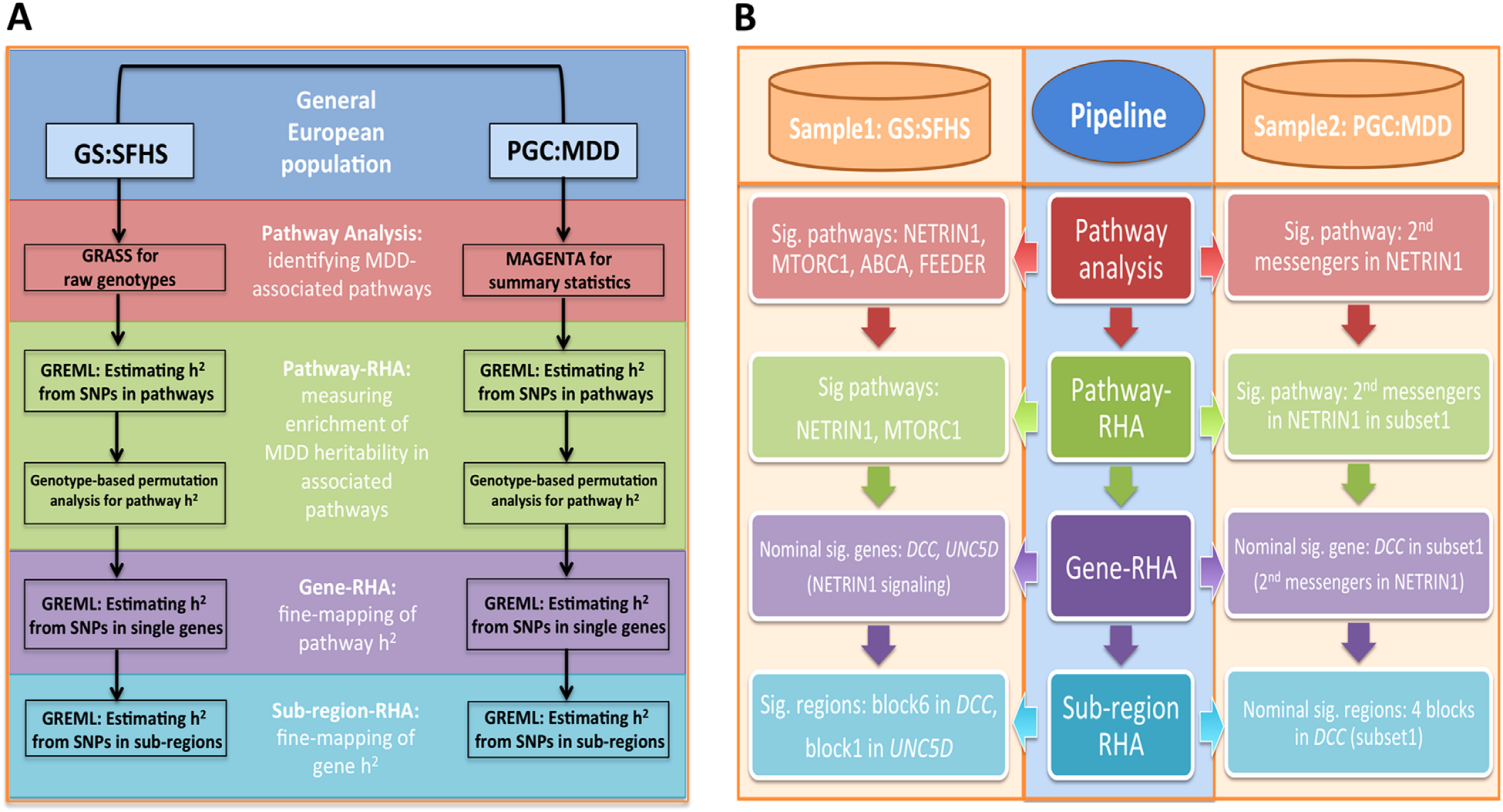
The analytical pipeline, its application in identifying associated pathways with major depressive disorder (MDD) and its findings in two independent samples: Generation Scotland: The Scottish Family Health Study (GS:SFHS) and Psychiatric Genomics Consortium (PGC:MDD). **(A)** Design of the analytical pipeline. In the pipeline, pathway analysis was performed for 1035 pathways, significant pathways were analyzed with multilevel regional heritability analyses (RHAs) in the framework of genomic restricted maximum likelihood (GREML) analysis to quantify and localize the genetic effects on MDD. In pathway analysis, GRASS was applied to the phenotype and genotype data of 6455 individuals from the GS:SFHS sample. MAGENTA was applied to the summary data from PGC:MDD genome-wide association studies (GWASs) on 18,759 individuals. **(B)** The findings by the analytical pipeline in the two samples. The NETRIN1 signaling pathway was identified as an associated pathway with MDD, and the association signals were localized to its gene *DCC* and the subregion level. *h*^2^, heritability; Sig, significant; SNP, single nucleotide polymorphism.

This pipeline includes two stages of analyses: a pathway analysis to identify MDD-associated pathways and multilevel (pathway/gene/subregion) regional heritability analyses to narrow the signals of the association. The multilevel regional heritability analyses for pathways identified by stage 1 were tested on the same sample in which they were first identified, and the test statistics from stage 2 are therefore potentially biased toward finding a more significant association. However, by applying the pipeline to two or more samples and by seeking replication across these the pipeline could provide independent replication of findings (as shown in the present study). Further details of the pipeline are shown in Figure 1 and Supplemental Table S2.

#### Stage 1: Pathway Analysis

For both samples, SNPs were annotated to 1035 pathways (640 from Reactome [http://www.reactome.org], 216 from BioCarta [http://cgap.nci.nih.gov/Pathways/BioCarta_Pathways], and 179 from Kyoto Encyclopedia of Genes and Genomes [http://www.genome.jp/kegg/]) (Supplement). For the GS:SFHS genotype data set (*n*_SNP_ = 592,690; *n*_sample_ = 6455), the GRASS (gene set ridge regression in association studies) algorithm (23) was used to identify pathway MDD associations using only the genotyped SNPs (Supplement). False discovery rate (FDR)-adjusted *p* values (*n*_FDR_ = 1035) were calculated with the function p.adjust in the R package “stats” (24, 25). For the PGC:MDD GWAS summary results data set (*n*_SNP_ = 1,074,100), MAGENTA (meta-analysis gene-set enrichment of variant associations (26) was used to test for the enrichment of genetic associations in each pathway for MDD, because only summary statistics were available for this part of the study (http://www.med.unc.edu/pgc/downloads), and MAGENTA was designed to exploit summary data from GWAS results (Supplement). MAGENTA reports a nominal *p* value and an estimated FDR per pathway (*n*_FDR_ = 1035).

#### Stage 2: Estimation of MDD Phenotypic Variance Explained by Imputed Genotypes of Regional SNPs (Regional Heritability)

Imputed SNPs were used in this analysis to avoid underestimating the regional heritability (12). For PGC:MDD, because of the heterogeneity caused by factors such as different ancestry and clinical diagnosis across samples (4) (Supplemental Table S1), as well as analyzing the regional heritability in the combined data set, we performed the SNP heritability analysis in the three subsets used by the PGC:MDD consortium to group the nine cohorts (27) (Supplemental Table S1).

We applied GREML with LMM (11) to estimate the variance explained by SNPs from genic regions of genes from candidate pathways and the subregions of candidate genes. A log-likelihood ratio test (LRT) was applied to test the significance of the estimated variance component (Supplement). Permutation analysis was performed to test whether the pathway-level regional heritability in candidate pathways was significantly greater than that expected by chance (Supplement). These analyses were performed in GCTA (11). To map regional heritability at the subregion level, regional heritability mapping (RHM; a modified GREML analysis) was applied using a sliding window to scan across the genic region of candidate genes (Supplement).

### Polygenic Risk Profiling

PRSs (28) estimate the genetic risk of MDD for unrelated individuals (*n*_sample_ = 6455) in GS:SFHS by adding the number of risk alleles an individual had, weighted by the effect size estimated in PGC:MDD (4) (Supplement).

To compare the PRS derived from the pathway SNPs to that derived from the whole-genome SNPs, logistic regression and LMMs were used to estimate the phenotypic variance explained by PRSs. For logistic regression, PRS was treated as a fixed effect and MDD phenotype was regressed on PRS (other covariates: age, age^2^, sex, top four principal components). The variance explained by PRS was calculated as Nagelkerke’s *R*^2^ on the observed scale (29). Permutation analysis was conducted to set an empirical threshold by creating PRS from 1000 circularly permuted SNP sets (30) that were then fitted in logistic regression. For LMM, we developed a PRS-bin-relationship matrix method in which the MDD phenotype variation was explained by the PRS similarity between subjects in the framework of LMM. The PRS similarity reflects relatedness in terms of the MDD genetic risk. The PRS similarity was treated as a random effect, and the variance explained by this random effect was estimated with REML and tested with LRT (Supplement).

Finally, the area under the receiver-operating characteristic curve (AUC) was calculated to determine the efficacy of PRSs in correctly classifying MDD case and control subjects using equation 2 in Wray *et al*. (31).

## Results

To identify candidate pathways for MDD, a pipeline that combines pathway and multilevel (pathway/gene/subregion) regional heritability analyses was applied to two independent samples, GS:SFHS and PGC:MDD, respectively. Detailed information of the analytical pipeline and the data usage is shown in Figure 1 and Supplemental Table S2.

### Identification of MDD-Associated Pathways in GS:SFHS

#### Pathway Analysis

By applying GRASS in GS:SFHS, four pathways were significantly associated with MDD after FDR correction (*n*_FDR_ = 1035). These comprised the following: three pathways from Reactome (MTORC1-mediated signaling, NETRIN1 signaling, ABCA transporters in lipid homeostasis) and one pathway from BioCarta (Feeder pathway) (Table 1).

#### Regional Heritability Analysis

With the use of GREML, the estimate of $h_{g}^{2}$(the heritability explained by all GWAS SNPs) for MDD was 0.25 (SE = 0.10) in GS:SFHS (Supplemental Table S4A). To further investigate the regional heritability captured by SNPs from pathways that were significant in pathway analysis, for each pathway, we partitioned the genome-wide SNPs into two sets as follows: SNPs from the pathway and the remaining SNPs. We then jointly estimated their contribution to MDD phenotypic variance in LMM. Among the four pathways that were significant in pathway analysis, two yielded significant *p* values (after FDR adjustment, *n*_FDR_ = 4) based on the LRT for pathway-level regional heritability in MDD, with the highest regional heritability estimated in the NETRIN1 signaling pathway [pathway-level regional heritability attributable to the pathway SNPs ($h_{R}^{2})$ = 0.014, SE = 0.009, *p*_*lrt_FDR*_ = .019] (Table 2). Permutation test across the circularly permuted SNP sets with the same set size for the two pathways showed that the detected pathway regional heritability was not attributable to gene set size and linkage disequilibrium (LD) structures (NETRIN1 signaling: *p*_*perm*_ = .018; MTORC1-mediated signaling pathway: *p*_*perm*_ = .01) (Supplement).

**Figure 2:**
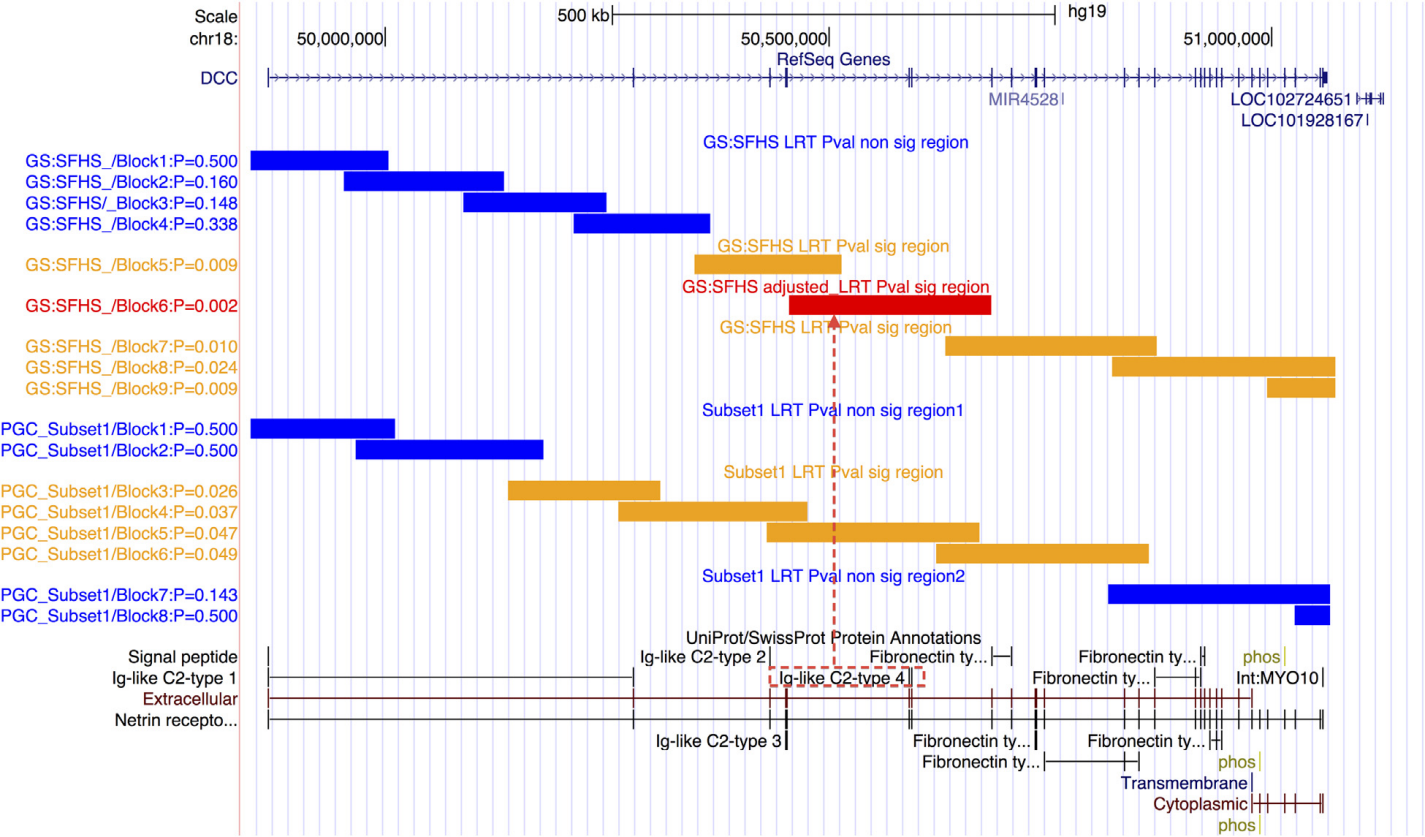
Genic region in *DCC* showing blocks used in REACTA on Generation Scotland: The Scottish Family Health Study (GS:SFHS) and subset 1 in Psychiatric Genomics Consortium Major Depressive Disorder (PGC:MDD) subset 1 (subsets 2 and 3 failed to obtained significance in the pathway-level regional heritability of NETRIN1 signaling pathway). In GS:SFHS, the sliding window [window size = 200 single nucleotide polymorphisms (SNPs)] defined 9 blocks with average block size of 179 kb. In PGC:MDD subset 1, window size of 100 SNPs was used, as the density of SNPs in PGC:MDD data set was approximately one half of that in GS:SFHS. This divided *DCC* into 8 blocks with average block size of 191 kb. Blue bar indicates insignificant region in log-likelihood ratio test (LRT); orange bar, significant region in LRT; red bar, significant region in LRT after Bonferroni correction; red dotted line, significant Block 6 in GS:SFHS, which overlaps with the fourth immunoglobulin-like (Ig) domain. This region was fully covered by the nominal significant regions in subset 1 in PGC:MDD.

To narrow the signals from the two pathways in which significant pathway-level regional heritability was detected, gene-level regional heritability was estimated for single genes in the two pathways. The heritabilities for three genes (*DCC*, *UNC5D*, and *SIAH2*) from the NETRIN1 pathway and one gene (*RPTOR*) from the MTORC1-mediated signaling pathway obtained nominal significance in LRT (Supplemental Table S5A). Among them, the receptor proteins encoded by *DCC* and *UNC5D* share the same ligand, Netrin-1, a key signaling molecule in the NETRIN signaling pathway (32). To fine-map the regional heritability within the two receptor genes and to further explore if any of the subregions that conferred heritability overlapped with any functional domains, RHM was applied to the two genes using a fixed sliding window to scan across their genic regions (Figure 2, Supplemental Figure S1). Block 6 in *DCC* and Block 1 in *UNC5D* yield significance in LRT (Block6_*DCC*_: *p*_*lrt_bonf*_ = .021; Block1_*UNC5D*_: *p*_*lrt_bonf*_ = .028) (Supplemental Table S6A). Block 6 in *DCC* overlapped with the fourth immunoglobulin-like domain (Figure 2) (33). Block 1 in *UNC5D* overlapped with H3K4me3 signal region (Supplemental Figure S1) (34).

### Identification of MDD-Associated Pathways in PGC:MDD

#### Pathway Analysis

With the use of MAGENTA, only one pathway from Reactome [role of second messengers in NETRIN1 signaling, which is a subset of the “the NETRIN1 signaling pathway” (100% overlap) (35)] was identified as associated with MDD after FDR correction (*n*_FDR_ = 1035) (Table 1).

#### Regional Heritability Analysis

Following the PGC published study, data from nine cohorts were grouped into three subsets for the GREML analysis (27) (Supplemental Table S1). The estimate of $h_{g}^{2}$ for MDD varied from 0.26 (SE = 0.06) to 0.47 (SE = 0.05) across subsets (Supplemental Table S4B). The pathway-level regional heritability from the role of second messengers in the NETRIN1 signaling pathway was nominally significant using LRT in subset 1 (*p*_*lrt*_ = .017, *p*_*lrt_FDR*_ = .07), whereas it was not significant in subset 2, subset 3, and the combined set (Table 2). Permutation test across the circularly permuted SNP sets with the same set size in subset 1 confirmed the enrichment of SNP heritability in this pathway (*p*_*perm*_ = .01) (Supplement). The gene-level regional heritability analysis for this pathway obtained nominal significance in one gene (*DCC*, *p*_*lrt*_ = .02) in subset 1, no genes in subset 2, one gene (*TRPC3*) in subset 3, and two genes (*PLCG1* and *PITPNA*) in the combined data set (Supplemental Table S5B). Because in subset 1 the pathway-level regional heritability was significant for the role of second messengers in the NETRIN1 signaling pathway and the gene-level regional heritability was significant in the *DCC* gene, we further localized the regional heritability by applying RHM to *DCC* in subset 1 (Supplemental Table S6B). The distribution of the regional heritability for the *DCC* gene in subset 1 was similar to that obtained in GS:SFHS (Figure 2).

### Replication of GS:SFHS Results in PGC:MDD and PGC:MDD Results in GS:SFHS

Among the four pathways identified by pathway analysis in GS:SFHS, the NETRIN1 signaling pathway was replicated in PGC:MDD (*p*_*path*_ = .010, *n*_bonf_ = 4 + 1 = 5, *p*_*path_bonf*_ = .05), whereas the other three pathways failed to replicate (Table 3). For the regional heritability analysis, among the two significant pathways in GS:SFHS, the NETRIN1 signaling pathway was significant in LRT in PGC:MDD subset 1 (*p*_*lrt*_ = .00258, *n*_bonf_ = 2 × (1 + 3) + 1 = 9, *p*_*lrt_bonf*_ = .02) but was not significant in other subsets or in the combined set (Table 4). The MTORC1-mediated signaling pathway failed to replicate in all subsets and in the combined set (Table 4). The gene-level regional heritability of *DCC* was nominally significant in both GS:SFHS (Supplemental Table S5A) and in subset 1 in PGC:MDD (Supplemental Table S5B). The significant block 6 of *DCC* that was detected in GS:SFHS was fully covered by the nominally significant blocks in subset 1 (Figure 2).

The only pathway identified by pathway analysis in PGC:MDD, the role of second messengers in NETRIN1 signaling pathway, was nominally significant in pathway analysis in GS:SFHS (*p*_*path*_ = .018, *n*_bonf_ = 5*, p*_*path_bonf*_ = .09) (Table 3). For the regional heritability analysis, this pathway was nominally significant in LRT in GS:SFHS (*p*_*lrt*_ = .017, *n*_bonf_ = 9, *p*_*lrt_bonf*_ = .156) (Table 4).

### Estimating the Predictive Accuracy of PRSs Derived From SNPs in the NETRIN1 Signaling Pathway or the Whole Genome

We applied polygenic risk profiling to measure the additive genetic effect from the NETRIN1 signaling pathway and compared it with that from the whole genome (Methods and Materials).

**Figure 3:**
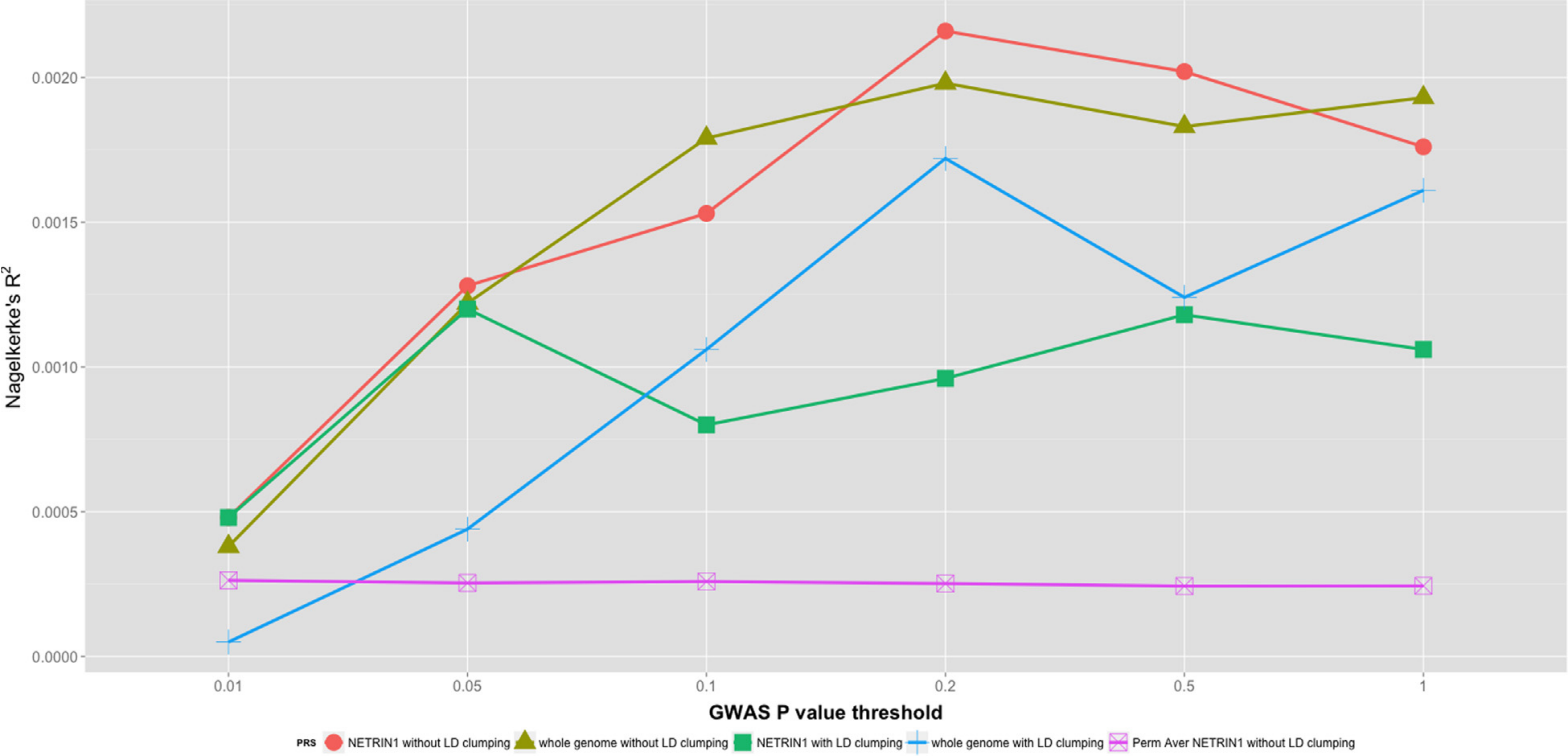
The phenotypic variance explained by polygenic risk score (PRS) as a fixed effect in logistic regression. Perm Aver NETRIN1 without linkage disequilibrium (LD) clumping: the average Nagelkerke’s *R*^2^ of 1000 PRSs created from permuted pathway single nucleotide polymorphisms (SNPs) (the circular permuted SNP sets with the same set size). GWAS, genome-wide association study.

With the use of logistic regression, for both pathway and whole-genome SNP sets, PRSs created without LD clumping explained a higher proportion of variance than PRSs created with LD clumping (Supplemental Table S7, Figure 3). The PRS created from the whole-genome SNPs explained a maximum MDD variance of 0.198% (GWAS *p*_*cutoff*_ = .2, without LD clumping; *p*_*t-test*_ =.006). The PRS created from SNPs in the NETRIN1 signaling pathway explained a maximum variance of 0.216% (GWAS *p*_*cutoff*_ = .2, without LD clumping; *p*_*t-test*_ = .004) (Supplemental Table S7). Permutation test across the circularly permuted SNP sets with the same set size suggested that the variance explained by the NETRIN1 signaling pathway PRS (without LD clumping) in the logistic regression model was significantly higher than expected by chance (Supplemental Table S8).

**Figure 4:**
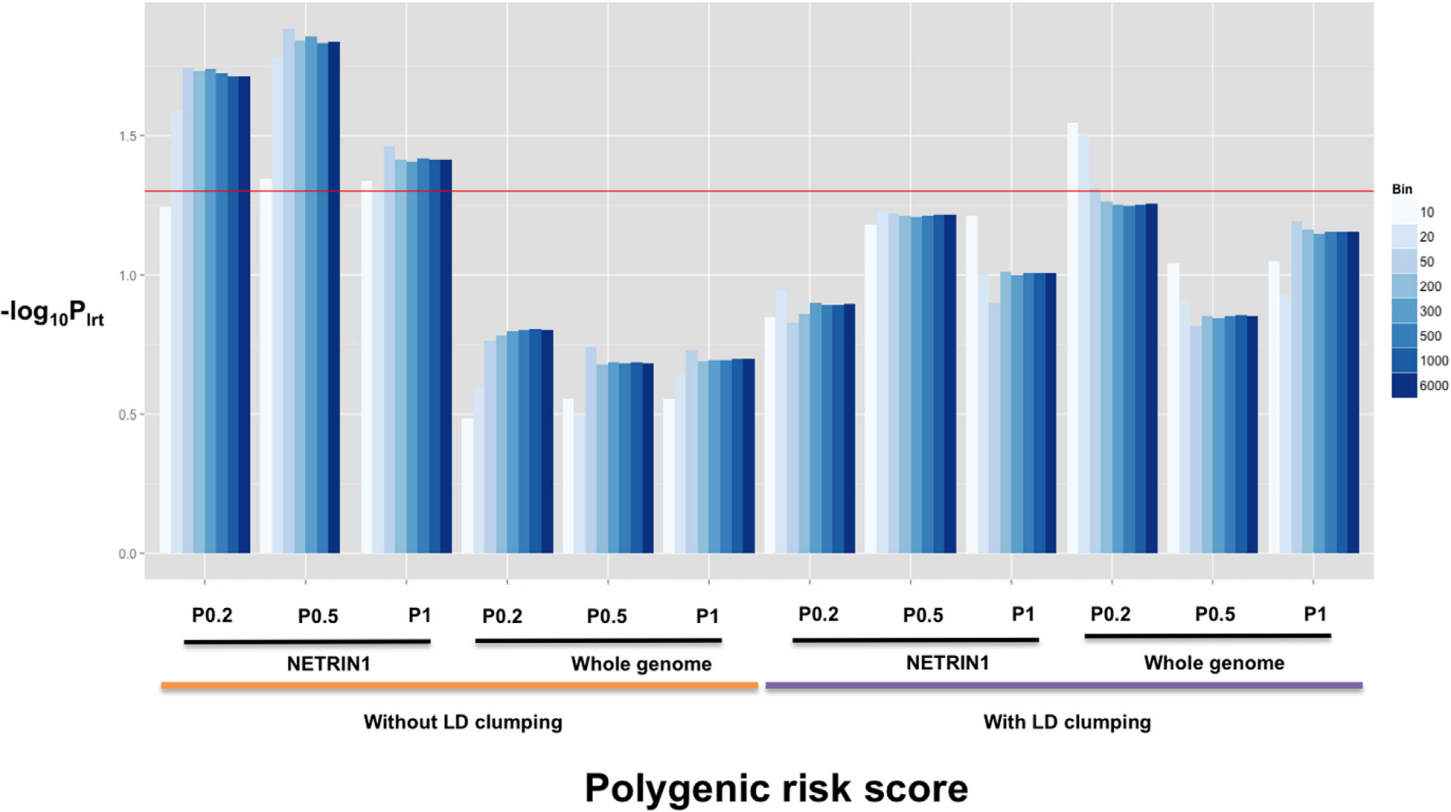
The log-likelihood ratio test (LRT) result from linear mixed modeling to show the significance level of the phenotypic variance explained by polygenic risk score-bin-relationship matrices derived from variants in whole genome and the NETRIN1 signaling pathway with or without linkage disequilibrium (LD) clumping at genome-wide association study *p* value thresholds of .2, .5, and 1, using different bins. The color of the bars was designated by the bin number. Red line marks the significance level (*p*_*lrt*_ = .05).

With the use of LMM, we estimated the proportion of MDD variance explained by pairwise MDD-PRS similarity between individuals. A PRS-bin-relationship variance-covariance matrix was constructed and jointly fitted with a SNP-based genomic-relationship matrix (GRM) in LMM. For each PRS, multiple bin numbers were tested to assess the stability of the model across different bin settings. When the comparison was between the PRSs created without LD clumping, the PRS-bin-relationship matrices created from the NETRIN1 signaling pathway outperformed those created from the whole-genome set because they consistently explained a significant proportion of variance across multiple bin settings and *p* value thresholds (Figure 4), with an explained maximum MDD variance of 1.7% (SE = 0.02, *p*_*lrt*_ = .013, *p*_*perm*_ = .012; PRS setting: bin = 50, GWAS *p*_*cutoff*_ = .5) (Supplemental Table S9). Nonetheless, for PRSs created with LD clumping, most of the PRS-bin-relationship matrices, created both from the whole-genome SNPs and from the pathway SNPs, failed to obtain significance in LRT, with three exceptions at low-bin-setting (bin = 10, 20, 50) for the whole-genome PRS-bin-relationship matrix (Figure 4), the maximum MDD variance explained is 2.47% (SE = 0.028, *p*_*lrt*_ = .028, *p*_*perm*_ = .021; PRS setting: bin = 10, GWAS *p*_*cutoff*_ = .2) (Supplemental Table S9). When jointly fitting PRS-bin-relationship matrices from the pathway and whole genomes as well as a GRM in LMM, the variance explained by the NETRIN1 pathway PRS (without LD clumping) remained stable and significant (Supplemental Table S10).

Finally, the estimation of the AUC receiver-operating characteristic suggested that in general the AUCs of MDD PRSs were low, ranging from 0.498 to 0.532, with the NETRIN1 PRS obtaining the highest AUC (AUC_NETRIN1_max_ = 0.532, AUC_whole_max_ = 0.527) (Supplemental Table S11).

## DISCUSSION

Typically, disease-associated pathways have been assumed to have the following features: 1) the genetic variants in them are shown to be associated with disease in association tests, 2) the genetic variants in them explain a significant proportion of phenotypic variance, and 3) the genetic proxies such as PRSs derived from them have valuable predictive power for the disease. Here, we applied a pipeline that integrates pathway analysis and regional heritability analyses to two independent samples. This enables the identification of candidate pathways for MDD that address the first two features. By comparing results from each stage of the pipeline, we identified the NETRIN1 signaling pathway, which has multilevel associations with MDD that are observed across samples. Finally, the polygenic risk profiling method provided additional evidence that this pathway also satisfied the third feature.

In the pathway analysis, we identified four MDD-associated pathways in GS:SFHS but only one in PGC:MDD by using two methods, GRASS and MAGENTA, respectively, because different data types were available from each data set for this analysis (Supplement). No pathway was associated with MDD in both GS:SFHS and PGC:MDD. However, the only associated pathway detected by MAGENTA in PGC:MDD, the role of second messengers in the NETRIN1 signaling pathway, was a subset of the NETRIN1 signaling pathway (100% overlap) that was detected by GRASS in GS:SFHS. Previous studies have suggested that the NETRIN1 signaling pathway plays a crucial role in axon guidance, a process that establishes precise brain circuits during the development of the central nervous system (36). Interestingly, in the development stage of the thalamus, the response of embryonic thalamocortical axons to the NETRIN1 signaling is modulated by serotonin signaling, a system that has been repeatedly implicated in the cause of MDD (37, 38). Given these convergent lines of research, NETRIN1 signaling is a promising candidate pathway for MDD.

The pathway analysis was followed by a pathway-level regional heritability analysis. We found that in both samples some of the MDD-associated pathways, including the two NETRIN1 signaling pathways, contributed significantly to explain MDD variance. Moreover, the pathway-level regional heritability estimated was greater than expected, given the SNP set size in these pathways (Table 2), suggesting an enrichment of heritability further supported by the permutation test. These results were consistent with previous studies reporting enrichment of heritability in functionally annotated regions (12, 13).

In the NETRIN1 signaling pathway, key proteins affect axon guidance; DCC is the key receptor for the attractive response to Netrin-1, whereas UNC5, alone or together with DCC, is associated with the repulsive response to Netrin-1 (36, 39). In our study, the gene-based regional heritability analyses suggested that *DCC* and *UNC5D* were among the most associated genes in GS:SFHS, which is consistent with their functional importance in the NETRIN1 signaling pathway. In PGC:MDD*, DCC* was the only gene that attained nominal significance (in subset 1, the only subset for which the pathway-level regional heritability from the candidate pathway is significant). When applying RHM to *DCC*, the regional heritability was localized to block 6 in GS:SFHS (for PGC:MDD subset 1 this region also obtains nominal significance) (Figure 2). Block 6 overlapped with the fourth immunoglobulin-like domain (Figure 2), which may be necessary for the axonal attraction mediated by Netrin-1 and draxin (40). A recent meta-analysis of GWASs for depressive symptom (*N* =180,886) reported that one locus (rs62100776) from the same gene *DCC* exceeded genome-wide significance (it includes subjects from PGC:MDD, but the major source of sample is from UK biobank [*n*_ukb_ = 105,739]) (41). This SNP is located in block 7, which is adjacent to the significant block 6 and is nominally significant in our study (Figure 2). This overlapped finding, using a much larger sample size, validated our results and indirectly supported the value of applying our pipeline in studies with small sample size. The significant block in *UNC5D* overlapped with an H3K4me3 modification, which implicates an active promoter in that region (42). These results imply a potentially functional contribution of variants in *DCC* and *UNC5D* to MDD.

The pathway identified with our pipeline accounts for a significant proportion of phenotypic variance, which is an attractive feature for a biomarker. PRSs were created by adding the genetic effects among biomarkers. Although MDD is a highly polygenic disorder, PRSs from whole-genome SNPs can be noisy because they include SNPs with no effect on MDD. A more accurate prediction is likely from scores derived from biomarkers with a higher proportion of causal SNPs. We thus measured the prediction value for MDD of the PRSs derived from the associated NETRIN1 signaling pathway. The results showed that pathway PRSs explain a higher proportion of phenotypic variance than whole-genome PRSs, when PRS was fitted as a fixed effect in the logistic regression. The AUC statistics also support a better prediction by the pathway PRSs. In addition, we developed a PRS-bin-relationship matrix method in which PRS similarity was used to explain phenotypic variation of MDD in LMM. With the use of this method, the MDD phenotypic variance explained by the pathway PRS and whole-genome PRS was substantially increased to 1.70% and 2.47%, respectively. Although the largest variance explained by the pathway PRS was smaller than the whole-genome PRS, the pathway PRS performed better in terms of the significance level in LRT across most of the tested bins (Figure 4).

Notably, although the PRS-bin-relationship matrix is conceptually similar to the classic common SNP-based GRM, a key difference was that the PRS-bin-relationship matrix took the information of the effect size of loci as estimated in the discovery sample, PGC:MDD, and the genotypes from the target sample, GS:SFHS, so that it measures the MDD genetic risk similarity and the interpretation of the model which it fitted was across samples. This method also enabled the discrimination of genetic effects represented in the pathway PRS, whole-genome PRS, and GRM when they were jointly fitted in LMM. Our results suggest that they explained distinct proportions of phenotypic variance (1.6% for pathway PRS, 2.2% for whole-genome PRS, and 22.7% for whole-genome GRM) (Supplemental Table S10).

With regard to limitations and further research, first, different methods were applied in different samples that used different data formats in the pathway analysis. This may have influenced the consistency and the comparability of the results and may have complicated their interpretation. Second, it is possible that the predictive accuracy of the NETRIN1 signaling pathway measured in this study is inflated, because both samples were involved in the pipeline where this pathway was identified (albeit independently); further replication in independent populations would strengthen our findings. Note that although the multilevel regional heritability analyses enable the fine-mapping of association signals, their *p* values may be inflated because the fine-mapping was conducted at the same data sets where the pathways were identified. We therefore suggest a careful interpretation of the LRT results from the regional heritability analyses, and we attach more importance to the comparisons of the regional heritability patterns across populations (Figure 2). Third, the route by which NETRIN1 signaling pathway contributes to MDD is unknown. Future directions for increasing our understanding could include exploring mutant DCC animal models of MDD and testing the interactions of NETRIN1 receptors with known MDD-associated proteins.

In summary, this study shows that the NETRIN1 signaling pathway was associated with MDD in two independent samples. Variants in this pathway accounted for a significant proportion of variance in susceptibility for MDD and have valuable prediction power. These findings further support a role for NETRIN1 in the cause of MDD and provide a basis for future studies.

## Figures and Tables

**Table 1:** Top 10 Pathways in Pathway Analysis for MDD Using GRASS on GS:SFHS and Using MAGENTA on PGC:MDD

Rank	Database	Pathway	*p* Value	*p*_FDR_	Eff Gene Size	Exp No. of Genes >95% Cutoff	Obs No. of Genes >95% Cutoff
GS:SFHS							
1	BioCarta	Feeder pathway	2.93E–05	2.14E–02^a^	—	—	—
2	Reactome	ABCA transporters in lipid homeostasis	5.20E–05	2.14E–02^a^	—	—	—
3	Reactome	NETRIN1 signaling	6.20E–05	2.14E–02^a^	—	—	—
4	Reactome	MTORC1-mediated signaling	8.87E–05	2.30E–02^a^	—	—	—
5	BioCarta	P35 Alzheimer’s pathway	2.69E–04	5.58E–02	–	—	—
6	BioCarta	SODD pathway	5.23E–04	8.59E–02	—	—	—
7	Reactome	Energy-dependent regulation of MTOR by LKB1 AMPK	7.46E–04	8.59E–02	—	—	—
8	Reactome	NFKB activation through FADD RIP1 pathway mediated by caspase 8 and 10	7.47E–04	8.59E–02	—	—	—
9	KEGG	Taste transduction	6.48E–04	8.59E–02	—	—	—
10	Reactome	ABC family protein-mediated transport	8.68E–04	8.98E–02	—	—	—
PGC:MDD							
1	Reactome	Role of second messengers in NETRIN1 signaling	1.00E–4	1.46E–02^a^	8	0	4
2	Reactome	Defensins	9.00E–04	2.04E–01	25	1	6
3	Reactome	NRAGE signals death through JNK	1.50E–03	2.07E–01	36	2	7
4	Reactome	β Defensins	3.50E–03	2.75E–01	21	1	5
5	Reactome	Purine catabolism	1.26E–02	3.11E–01	10	1	3
6	Reactome	Formation of tubulin folding intermediates by CCT TRIC	1.71E–02	6.67E–01	22	1	4
7	BioCarta	AKAP13 pathway	1.88E–02	7.31E–01	12	1	3
8	Reactome	Chondroitin sulfate dermatan sulfate metabolism	5.54E–02	7.41E–01	49	2	5
9	Reactome	Elevation of cytosolic CA2 levels	5.67E–02	7.43E–01	10	0	2
10	Reactome	Opsins	4.30E–02	7.46E–01	10	0	2

*n*_FDR_ = 1035.

Eff, effective; Exp No. Genes >95% Cutoff, expected number of genes with a corrected gene *p* value >95 percentile enrichment cutoff; FDR, false discovery rate; GS:SFHS, Generation Scotland: The Scottish Family Health Study; KEGG, Kyoto Encyclopedia of Genes and Genomes; MDD, major depressive disorder; Obs No. Genes >95% Cutoff, observed number of genes with a corrected gene *p* value >95th percentile enrichment cutoff; PGC, Psychiatric Genomics Consortium.

a For GRASS results on GS:SFHS, four pathways yielded significance after FDR correction. For MAGENTA results on PGC:MDD, one pathway yielded significance after FDR correction.

**Table 2:** Pathway-Level Regional Heritability Analysis Results for the Significant Pathways Identified in Pathway Analysis for GS:SFHS and PGC:MDD

Pathway or Group	*h*^*2*^_R_	SE (*h*^*2*^_R_)	*h*^*2*^_C_	SE(*h*^*2*^_C_)	LRT (*h*^*2*^_R_) *p* Value	LRT (*h*^*2*^_R_) *p*_FDR_	*n*_pathway SNPs_	%SNP	%*h*^*2*^_GWAS_	%*h*^*2*^_GWAS_/%SNP
GS:SFHS										
Reactome MTORC1-mediated signaling	0.006	0.004	0.240	0.099	7.70E–03	1.86E–02^a^	947	0.04	2.40	54.84
Reactome NETRIN1 signaling	0.014	0.009	0.224	0.099	9.28E–03	1.86E–02^a^	8809	0.41	5.80	14.20
BioCarta feeder pathway	0.004	0.004	0.251	0.099	3.77E–02	5.03E–02	507	0.02	1.00	42.68
Reactome ABCA transporters in lipid homeostasis	0.000	0.004	0.251	0.099	5.00E–01	5.00E–01	1020	0.05	0.00	0.00
PGC:MDD: Role of Second Messengers In NETRIN1 Signaling										
PGC:MDD combined	0.0001	0.0006	0.2616	0.0216	3.77E–01	4.17E–01	1083	0.001	0.0005	0.52
PGC:MDD subset 1	0.0055	0.0034	0.2625	0.0596	9.06E–03	3.62E–02^a^	1083	0.001	0.0193	19.12
PGC:MDD subset 2	0.0010	0.0018	0.4655	0.0525	2.56E–01	4.17E–01	1083	0.001	0.0034	3.37
PGC:MDD subset 3	0.0004	0.0022	0.4442	0.0780	4.17E–01	4.17E–01	1083	0.001	0.0014	1.41

FDR, false discovery rate; GS:SFHS, Generation Scotland: The Scottish Family Health Study; GWAS, genome-wide association study; $h_{C}^{2},$ heritability attributable to the complement SNP set; $h_{R}^{2}$, pathway-level regional heritability attributable to the pathway SNPs; LRT, log-likelihood ratio test; LRT (*h*^*2*^_R_) *p* value and LRT (*h*^*2*^_R_) *p*_FDR_, nominal *p* value and FDR-adjusted *p* value from LRT for $h_{R}^{2}$; *n*_pathway SNPs_, SNP number in the pathway; PGC:MDD, Psychiatric Genomics Consortium Major Depressive Disorder; SNP, single nucleotide polymorphism; %$h_{\mathrm{GWAS}}^{2}$/%SNP, the ratio of the percentage of $h_{\mathrm{gwas}}^{2}$ in pathway to the percentage of SNPs in the pathway.

a Significant results after multiple test correction (5%).

**Table 3:** Replication Results of Pathway Analysis

Database	Pathway	*p* Value	*p*_bonf_	Eff Gene Size	Exp No. Genes >95% Cutoff	Obs No. Genes >95% Cutoff
GS:SFHS in PGC:MDD						
Reactome	ABCA transporters in lipid homeostasis	5.39E–01	1.00E+00	15	1	1
Reactome	NETRIN1 signaling	9.90E–03	4.95E–02^a^	37	2	6
Reactome	MTORC1-mediated signaling	4.05E–01	1.00E+00	10	1	1
BioCarta	Feeder pathway	1.00E+00	1.00E+00	9	0	0
PGC:MDD in GS:SFHS						
Reactome	Role of second messengers in NETRIN1 signaling	1.75E–02	8.76E–02	—	—	—

Four pathways were identified from GS:SFHS in PGC:MDD, and one pathway was identified from PGC:MDD in GS:SFHS. *n*_bonf_ = 5.

Eff, effective; Exp No. Genes >95% Cutoff, expected number of genes with a corrected gene *p* value >95th percentile enrichment cutoff; GS:SFHS, Generation Scotland: The Scottish Family Health Study; Obs No. Genes >95% Cutoff, observed number of genes with a corrected gene *p* value >95th percentile enrichment cutoff; *p*_*bonf*,_ adjusted *p* value using Bonferroni multiple testing correction; PGC:MDD, Psychiatric Genomics Consortium Major Depressive Disorder.

a Significant results after multiple test correction (5%).

**Table 4:** Replication Results of Pathway-Level Regional Heritability Analysis

	NETRIN1 Signaling						MTORC1-Mediated Signaling					
	*h*^*2*^_R_	SE (*h*^*2*^_R_)	*h*^*2*^_C_	SE (*h*^*2*^_C_)	LRT (*h*^*2*^_R_) *p* Value	LRT (*h*^*2*^_R_) *p*_bonf_	*h*^*2*^_R_	SE (*h*^*2*^_R_)	*h*^*2*^_C_	SE (*h*^*2*^_C_)	LRT (*h*^*2*^_R_) *p* Value	LRT (*h*^*2*^_R_) *p*_bonf_
GS:SFHS in PGC:MDD: Group												
Combined	0.000	0.001	0.262	0.022	4.75E–01	1.00E+00	0.0003	0.0005	0.2843	0.0220	2.71E-01	1.00E+00
Subset 1	0.014	0.006	0.256	0.059	2.59E–03	2.33E–02^a^	0.0006	0.0015	0.2647	0.0597	3.32E-01	1.00E+00
Subset 2	0.003	0.004	0.463	0.052	1.59E–01	1.00E+00	0.0018	0.0019	0.4643	0.0524	1.02E-01	9.17E–01
Subset 3	0.002	0.005	0.443	0.078	3.83E–01	1.00E+00	0.0000	0.0018	0.4447	0.0781	5.00E-01	1.00E+00
PGC:MDD in GS:SFHS: Pathway												
Reactome role of second messengers in NETRIN1 signaling	—	—	—	—	—	—	0.005	0.004	0.235	0.099	1.73E-02	1.56E–01

Two pathways were significant in GS:SFHS in PGC:MDD, and one pathway was significant in PGC:MDD in GS:SFHS. *n*_bonf_ = 9.

GS:SFHS, Generation Scotland: The Scottish Family Health Study; $h_{C}^{2},$ heritability attributable to the complement SNP set; $h_{R}^{2}$, pathway-level regional heritability attributable to the pathway SNPs; LRT, log-likelihood ratio test; LRT (*h*^*2*^_R_) *p*_*bonf*_, adjusted *p* value from LRT for $h_{R}^{2}$ using Bonferroni multiple testing correction; LRT (*h*^*2*^_R_) *p* value, nominal *p* value from LRT for $h_{R}^{2}$; PGC:MDD, Psychiatric Genomics Consortium Major Depressive Disorder.

a Significant results after multiple test correction (5%).
