# Supplementary material for: A Combined Pathway and Regional Heritability Analysis Indicates NETRIN1 Pathway Is Associated With Major Depressive Disorder
Source: Biol Psychiatry. 2017 Feb 15;81(4):336–46. doi: 10.1016/j.biopsych.2016.04.017 (PMC5262437; doi:10.1016/j.biopsych.2016.04.017)
Supplement: Supplementary file 1 — Supplementary material [file mmc1.pdf]

# A Combined Pathway and Regional Heritability Analysis Indicates NETRIN1 Pathway is Associated with Major Depressive Disorder

## *Supplementary Information*


MDD Diagnosis, Heterogeneity and Its Potential Influence on the Current Study ............................... 2

Quality Control and Imputation in GS:SFHS ........................................................................ 3

SNP Annotation and Pathway Analysis .................................................................................. 3

Regional Heritability Analysis ............................................................................................... 4

Permutation Analysis for Regional Heritability ..................................................................... 5

Regional Heritability Mapping .............................................................................................. 6

Polygenic Risk Scoring .......................................................................................................... 6

PRS-bin Relationship Matrix Methods .................................................................................. 7

Table S1. Details of the nine primary cohorts and the grouping of sub-samples in the PGC:MDD dataset. ................................................................................................................................ 8

Table S2. Data usage information of the application of the pipeline in the two samples ....................... 9

Table S3. RHM results for *DCC* and *UNC5D* using different window sizes in GS:SFHS ................. 10

Table S4. SNP heritability (the heritability explained by all the imputed SNPs) for MDD ................ 13

Table S5. Gene-regional heritability attributable to genes in the pathway that had significant pathway-level regional heritability ............................................................................................. 14

Table S6. RHM result for *DCC* and *UNC5D*. ............................................................................. 18

Table S7. Comparison of the variance explained by the fixed effect from NETRIN1 signaling PRS and whole genome PRS, and the *t* test result of the effect in logistic regression. ................................ 19

Table S8. Permutation result for NETRIN1 PRS (without LD clumping) in logistic regression. ........ 19

Table S9. Comparison of the variance explained by the random effect from the NETRIN1 signaling PRS and whole genome PRS in LMM. ........................................................................................ 20

Table S10. Phenotypic variance attributable to the three variance components by jointly fitting NETRIN1 signaling PRS-bin relationship matrix, whole genome PRS-bin relationship matrix and genomic relationship matrix in LMM. ........................................................................................ 22

Table S11. AUC statistics of PRSs. ............................................................................................. 23

Figure S1. Genic region in *UNC5D* showing blocks used in REACTA in GS:SFHS .......................... 24

Description of the Symbols Used in the Main Text ..................................................................... 25

Supplemental References ............................................................................................................ 26

## MDD Diagnosis, Heterogeneity and Its Potential Influence on the Current Study

*GS:SFHS*

Participants who screened positive for a mood disorder (21.7%) were invited to continue to an interview using the SCID modules for mood disorders (1). Participants who screened positive for a mood disorder but refused to undergo the structured clinical interview ($n$ = 507) and those with a diagnosis of bipolar disorder ($n$ = 76) were excluded from this study.

*PGC:MDD*

For the nine cohorts, either structured diagnostic interviews or clinical diagnosis were applied for the diagnosis. For most cohorts, cases were diagnosed from clinical sources (Table S1) (2). Two cohorts (GSK and RADIANT) require recurrent MDD and one cohort requires recurrent, early onset MDD (GenRED) (Table S1) (2). Most controls were screened for lifetime history of MDD and randomly selected from the matched population (Table S1) (2).

*Heterogeneity and Potential Influence on Study*

The factors such as different sources of diagnosis for cases and controls (clinical or population), different requirement of diagnosis (single episode or recurrent, early-onset or lifetime) and different genetic background (population) are potential contributors for heterogeneity. We have also observed that the estimates of the total GWAS SNP-heritability of MDD varied widely across subsets in PGC:MDD (Table S4B). These factors, alongside published evidence of widespread clinical heterogeneity (3), suggests the presence of genetic heterogeneity between populations, and across the PGC:MDD cohorts. These factors made it difficult to completely replicate results across datasets. The important implication from this study, however, is that even given the existing difference in clinical diagnosis (Table S1) and the intrinsic genetic heterogeneity of MDD (3), by applying the pipeline that combining the results from multiple analyses across population and across molecular level (in this study, pathway, gene and sub-region), constant signals from the NETRIN1 signaling pathway were observed across samples in MDD.

**Quality Control and Imputation in GS:SFHS**

Quality control of genotyped SNPs used inclusion thresholds: missing SNPs per individual ≤2%, SNP genotype call rate ≥98%, minor allele frequency >1% and Hardy-Weinberg equilibrium *P* value > $1 \times 10^{-6}$. Genotype data were also imputed from the 1000 genomes reference panel (Phase I integrated release v3, April 2012) using IMPUTE2 (4,5) after using SHAPEIT v2 (6) for pre-phasing the genotype data. Only high-quality imputed genotypes remained after removing those SNPs with an $R^2$ score (a measure for imputation quality) less than .6. The best-guess imputed genotypes were then generated and accepted at the same quality control levels as for the genotyped SNPs, and were used in whole-genome and regional heritability analysis.

**SNP Annotation and Pathway Analysis**

SNPs were assigned to a genic region if the SNP was located between 20 kb upstream of the transcription start site and 20 kb downstream of the transcription end site (7), as 95% of eQTLs lie within 20 kb upstream of the TSS (8). Pathways were downloaded from the Molecular Signatures Database v4.0 (9). Two filtering steps were applied to the pathways: first, pathways that contained less than 5 genes or more than 200 genes were removed to avoid false positive associations due to large single-gene or single-SNP effects in small gene-sets, or the association by chance alone in large gene-sets (10). Second, pathways were removed for which less than 70% of genes were found in the NCBI gene database. All of the annotation steps were conducted using ANNOVAR (11). By the date of publication, the analyses presented in the current manuscript are the only ones that used GS:SFHS data to identify MDD-associated pathways.

*The Selection of the Tools Applied for Each Dataset*

The methods selected depend on the data type available and the research goals. GRASS was applied to raw GS:SFHS genotype and phenotype data as there is no GWAS published as yet for this dataset and hence summary statistics are not available. MAGENTA was applied to PGC:MDD as only the summary statistics of PGC:MDD were initially available to us when we performed the pathway analysis. We later obtained the raw genotypes for each cohort, but with the permission for the regional

heritability analyses only. The strategy of combining analyses of raw data from some studies with summary statistics from others was shown to be successful in identifying pathways and genes associated with schizophrenia using results from three independent samples (7). Furthermore, heterogeneity existed across the nine cohorts (as discussed above) and pathway analysis that takes the raw genotype as input, could have introduced stratification that would bias the association test. On the other hand, performing analyses on the single PGC:MDD cohort (small to modest sample sizes, ranging from 942 to 3,254) would have attenuated the statistical power for pathway analysis.

*GRASS*

Missing genotypes for those SNPs were replaced by best-guess imputed genotypes. GRASS assesses gene set association by performing regularized logistic regression, accounting for multiple hits and/or LD in the same gene. In the regularized logistic regression model, SNP dosage was coded as 0, 1 or 2. Representative eigen SNPs for each gene were selected and their joint association with MDD risk was assessed. MDD case/control status was permuted 1,000 times to estimate the significance threshold for significance (12).

*MAGENTA*

After assigning SNPs to genes, corrected gene-association scores were created by assigning the most significant GWAS *P* value of all SNPs that lie within the extended 20 kb boundaries of each gene and adjusting for potential confounders such as SNP number per kb, LD and gene size. Gene scores were combined to create pathway scores. For each pathway, an enrichment *P* value was created by a non-parametric statistical test which tests whether the gene-association score for the genes in a pathway are enriched for highly ranked scores (5%) to a greater extent than that would be expected by chance (13).

**Regional Heritability Analysis**

The variance explained by SNPs in a genomic region was estimated by jointly fitting a regional genomic relationship matrix (rGRM) and a complement genomic relationship matrix (cGRM) from all SNPs that are not included in creating rGRM in LMM.

$$Y = Xb + g_R + g_C + e$$

$$\text{Var}(Y_{random\_effect}) = A_R\sigma_R^2 + A_C\sigma_C^2 + I\sigma_e^2$$

$$h_R^2 = \sigma_R^2/\sigma_{Y\ random\_effect}^2$$

Where $\boldsymbol{Y}$ was a vector of MDD binary phenotypes, and $\boldsymbol{b}$ was a vector of covariates fitted as fixed effects (i.e., age, age$^2$, sex, 20 principal components derived from the genome-wide GRM). Principal components were calculated for unrelated individuals using principal component analysis. $\boldsymbol{g_R}$ and $\boldsymbol{g_C}$ were the random genetic effects from the regional SNPs (in this study, the SNPs that were annotated to all genes, single genes or sub-regions in genes in a specific pathway) and the complement set of SNPs, respectively. $\boldsymbol{A_R}$ and $\boldsymbol{A_C}$ were the GRMs created from the regional SNPs and the complement set of SNPs, respectively. The variance explained by each variance component including the regional heritability $\boldsymbol{h_R^2}$ was estimated using restricted estimated maximum likelihood (REML). The estimate was transformed from the observed scale to the liability scale assuming MDD prevalence of 0.13. A log likelihood ratio test (LRT) was applied to test the significance of each random effect by comparing a model with an rGRM fitted against a model without an rGRM.

**Permutation Analysis for Regional Heritability**

In order to test whether the pathway-level regional heritability in MDD-associated pathways was significantly greater than that is expected by chance, a circular genomic permutation method was applied to the SNP genotypes to test whether the pathway-level regional heritability was higher than the heritability explained by a random set of SNPs of the same number while accounting for the LD structure of the original SNP set (14). The regional heritability of permuted SNP sets was estimated by REML in GCTA. LRT statistics for the proportion of variance explained by the regional GRM were used to construct empirical distribution, from which permuted *P* values for the regional heritability from the pathway of interest were calculated.

## Regional Heritability Mapping

In the previous analyses of regional heritability analysis, we identified candidate pathways that contributed significantly to the MDD heritability at the pathway and gene level. To further narrow down the genomic regions in genes from the candidate pathway conferring the regional heritability of MDD, regional heritability mapping (RHM) was performed in REACTA (15), a modified version of GCTA, which enables a sliding window with fixed size (containing a constant number of SNPs) to scan the variance explained by the SNPs across a pre-defined genomic region. Here, we applied this method to single candidate genes. Best-guess imputed genotypes were used here for consistency. In GS:SFHS, we used 9 consecutive windows of size 200 SNPs and overlap of 20 SNPs to detect regional heritability across the genic region (containing 20 kb extended regions upstream and downstream of gene boundaries) of candidate genes. The results from RHM using other window sizes are shown in Table S3. For PGC:MDD, 100 SNP windows were used as the density of SNPs in PGC:MDD dataset was around half of that in GS:SFHS. This divided genic regions to 8 window blocks. As in the pathway-level and gene-level regional heritability analyses, an LRT was conducted to compare a full model with a block (window) fitted against the null model without that block (window) and REML estimated the proportion of variance explained by the block (window). The number of total windows was used to obtain a Bonferroni corrected threshold. The UCSC genome browse r(16) was used for genome annotation and visualization of the blocks.

## Polygenic Risk Scoring

Separate polygenic risk scores were created for GS:SFHS at GWAS *P* value thresholds of .01, .05, .1, .2, .5 and 1 by using the genotyped SNPs from 1) the NETRIN1 signaling pathway and 2) the whole genome. LD (linkage disequilibrium) clumping is usually adopted to create SNP-sets in linkage equilibrium for PRS by selecting SNPs that are in linkage equilibrium and are significant at a pre-defined GWAS *P* value threshold within a window. However this excludes signals from removed SNPs and may influence the accuracy of prediction (17). We therefore created the PRSs with LD clumping (window size = 300 kb; $r^2 \leq .2$) and without LD clumping at each GWAS *P* value threshold.

All the PRSs were created in the software PRSICE (18) as it provided high-resolution (for multiple GWAS *P* value thresholds) PRSs using the profiling function in PLINK in batch mode (19).

**PRS-bin Relationship Matrix Methods**

The variance explained by the PRS as a random effect was estimated by using the formula:

$$Y = X\beta + R_{PRS} + g + e$$

$$\mathrm{Var}(Y_{random\_effect}) = A_{PRS}\sigma^2_{PRS} + A_g\sigma^2_g + I\sigma^2_e$$

$$h^2_{PRS} = \sigma^2_{PRS}/\sigma^2_{Y\ random\_effect}$$

***Y*:** MDD binary phenotypes. ***β***: covariates (i.e., age, age$^2$, sex and 20 principal components). $\boldsymbol{R_{PRS}}$: the random effect from the PRS. $\boldsymbol{A_{PRS}}$: the PRS-bin relationship matrix. To create $\boldsymbol{A_{PRS}}$, PRSs for all individuals were ranked according to their PRS in an ascending order. After setting the total number of bins, N, each PRS was assigned a bin number. The pairwise coefficient between two individuals was then calculated based on the bin difference between them:

$$A_{ij(PRS)} = 1 - \mathrm{abs}\ (\ bin_{i(PRS)} - bin_{j(PRS)}\ )\ /\ N$$

***g*:** the random genetic effects from all the SNPs . $\boldsymbol{A_g}$**:** the GRM created from whole-genome SNPs (i.e., the standard whole-genome GRM in GCTA). The REML function in GCTA was used to estimate each variance component and LRT *P* values were used to test significance. To assess the LRT *P* value, a permutation test was performed to generate a null distribution by creating PRSs after permuting SNP ids across original SNP effect sizes 1000 times.

**Table S1. Details of the nine primary cohorts and the grouping of sub-samples in the PGC:MDD dataset.**

| Sample | Place | N_id | N_cases | N_controls | Def | Case Source | Control Source | % Case |
|---|---|---|---|---|---|---|---|---|
| GAIN | NL | 3,254 | 1,605 | 1,649 | MDD | clin/pop | pop | 0.49 |
| GenRED | US | 2,519 | 947 | 1,572 | reoMDD | vol | pop | 0.38 |
| GSK | GER | 1,748 | 889 | 859 | rMDD | clin | pop/clin | 0.51 |
| MDD2000-QIMR_610 | Mix AUS | 1,154 | 427 | 727 | MDD | pop | pop | 0.37 |
| MDD2000-QIMR_317 | Mix AUS | 1,913 | 990 | 923 | MDD | pop | pop | 0.52 |
| MPIP | GER | 942 | 405 | 537 | MDD | clin | pop | 0.43 |
| Bonn/Mann | GER | 2,204 | 928 | 1,276 | MDD | clin | pop | 0.42 |
| RADIANT | MIX | 3,138 | 1,591 | 1,547 | rMDD | clin | pop/vol | 0.51 |
| STAR*D | US | 2,068 | 1,193 | 875 | MDD | clin | pop | 0.58 |

| Group | Place | N_id | N_cases | N_controls | Def | Case Source | Control Source | % Case |
|---|---|---|---|---|---|---|---|---|
| Subset1 (GAIN+MDD2000-QIMR_610+MDD2000-QIMR_317) | Mixed | 6,229 | 2,990 | 3,239 | MDD | clin/pop | pop | 0.48 |
| Subset2 (RADIANT+GenRED+STAR*D) | Mixed | 6,920 | 3,230 | 3,690 | Mixed | clin/vol | pop/vol | 0.47 |
| Subset3 (Bonn/Mann+MPIP+GSK) | GER | 4,792 | 2,130 | 2,662 | Mixed | clin | pop/clin | 0.45 |
| Combined (all samples) | Mixed | 17,845 | 8,793 | 9,052 | Mixed | cin/pop/vol | pop/clin | 0.49 |

N_total: number of subjects. N_cases: number of cases. N_controls: number of controls. Def: definition of MDD. rMDD: recurrent MDD. reoMDD: recurrent, early age of onset MDD. % Cases: proportion of cases in the total sample. Clin: clinical ascertainment. Pop: population-based ascertainment. Vol: volunteer sample.

**Table S2. Data usage information of the application of the pipeline in the two samples.**

| | GS:SFHS ($t \leq 0.025$) | | PGC MDD ($t \leq 0.025$) | | | |
|---|---|---|---|---|---|---|
| | Genotype + Phenotype | | GWAS Summary Statistics | | Genotype + Phenotype | |
| | **Information Used** | **Method** | **Information Used** | **Method** | **Information Used** | **Method** |
| Pathway analysis | | GRASS | GWAS *P* value | MAGENTA | NA | NA |
| Regional heritability | Allele dosage, phenotype | GREML for :<br>pathway-level $h^2_R$ (GCTA)<br>gene-level $h^2_R$ (GCTA)<br>sub-region level $h^2_R$ (REACTA) | NA | NA | Allele dosage, phenotype | GREML for :<br>pathway-level $h^2_R$ (GCTA)<br>gene-level $h^2_R$ (GCTA)<br>sub-region level $h^2_R$ (REACTA) |
| Polygenic risk profiling | | Polygenic risk score | SNP effect size | Polygenic risk scoring | NA | NA |

**Table S3. RHM results for *DCC* and *UNC5D* using different window sizes in GS:SFHS.**

| Gene | W Size | Chr | Start_pos | End_pos | $h^2_R$ | SE ($h^2_R$) | $h^2_C$ | SE ($h^2_C$) | LRT ($h^2_R$) *P* Value | LRT ($h^2_R$) $P_{bonf}$ |
|---|---|---|---|---|---|---|---|---|---|---|
| *DCC* | 100 | 18 | 49847408 | 49907296 | 0.0000 | 0.0022 | 0.2436 | 0.0991 | 5.00E-01 | 1.00E+00 |
| *DCC* | 100 | 18 | 49896109 | 49982791 | 0.0019 | 0.0030 | 0.2526 | 0.0993 | 3.13E-01 | 1.00E+00 |
| *DCC* | 100 | 18 | 49961055 | 50051374 | 0.0010 | 0.0019 | 0.2496 | 0.0993 | 2.22E-01 | 1.00E+00 |
| *DCC* | 100 | 18 | 50039676 | 50128294 | 0.0012 | 0.0022 | 0.2497 | 0.0993 | 2.26E-01 | 1.00E+00 |
| *DCC* | 100 | 18 | 50115886 | 50192989 | 0.0011 | 0.0017 | 0.2493 | 0.0993 | 1.41E-01 | 1.00E+00 |
| *DCC* | 100 | 18 | 50181192 | 50248759 | 0.0005 | 0.0012 | 0.2471 | 0.0993 | 2.48E-01 | 1.00E+00 |
| *DCC* | 100 | 18 | 50232913 | 50330763 | 0.0001 | 0.0010 | 0.2505 | 0.0994 | 4.68E-01 | 1.00E+00 |
| *DCC* | 100 | 18 | 50307270 | 50370920 | 0.0005 | 0.0015 | 0.2470 | 0.0994 | 3.71E-01 | 1.00E+00 |
| *DCC* | 100 | 18 | 50361058 | 50446555 | 0.0031 | 0.0032 | 0.2339 | 0.0992 | 1.11E-02 | 1.78E-01 |
| *DCC* | 100 | 18 | 50434994 | 50534989 | 0.0024 | 0.0024 | 0.2365 | 0.0993 | 8.24E-03 | 1.32E-01 |
| *DCC* | 100 | 18 | 50517776 | 50631316 | 0.0039 | 0.0035 | 0.2338 | 0.0992 | 4.42E-03 | 7.07E-02 |
| *DCC* | 100 | 18 | 50620708 | 50714232 | 0.0031 | 0.0032 | 0.2321 | 0.0992 | 4.42E-03 | 7.07E-02 |
| *DCC* | 100 | 18 | 50691838 | 50825620 | 0.0032 | 0.0032 | 0.2329 | 0.0992 | 1.10E-02 | 1.76E-01 |
| *DCC* | 100 | 18 | 50810506 | 50916342 | 0.0022 | 0.0028 | 0.2342 | 0.0993 | 5.87E-02 | 9.39E-01 |
| *DCC* | 100 | 18 | 50900432 | 51053176 | 0.0045 | 0.0051 | 0.2322 | 0.0992 | 1.72E-02 | 2.75E-01 |
| *DCC* | 100 | 18 | 50994208 | 51071812 | 0.0026 | 0.0027 | 0.2335 | 0.0993 | 9.01E-03 | 1.44E-01 |
| *DCC* | 50 | 18 | 49847408 | 49879058 | 0.0000 | 0.0030 | 0.2126 | 0.0981 | 5.00E-01 | 1.00E+00 |
| *DCC* | 50 | 18 | 49869965 | 49895738 | 0.0000 | 0.0032 | 0.2106 | 0.0980 | 5.00E-01 | 1.00E+00 |
| *DCC* | 50 | 18 | 49883150 | 49920216 | 0.0004 | 0.0015 | 0.2255 | 0.0985 | 3.90E-01 | 1.00E+00 |
| *DCC* | 50 | 18 | 49901564 | 49939739 | 0.0008 | 0.0019 | 0.2252 | 0.0984 | 3.69E-01 | 1.00E+00 |
| *DCC* | 50 | 18 | 49929623 | 49966318 | 0.0000 | 0.0031 | 0.2239 | 0.0984 | 5.00E-01 | 1.00E+00 |
| *DCC* | 50 | 18 | 49952593 | 50002997 | 0.0005 | 0.0018 | 0.2227 | 0.0984 | 4.33E-01 | 1.00E+00 |
| *DCC* | 50 | 18 | 49982880 | 50031120 | 0.0013 | 0.0021 | 0.2230 | 0.0984 | 1.62E-01 | 1.00E+00 |
| *DCC* | 50 | 18 | 50014454 | 50051374 | 0.0010 | 0.0018 | 0.2228 | 0.0984 | 2.25E-01 | 1.00E+00 |
| *DCC* | 50 | 18 | 50039676 | 50077817 | 0.0003 | 0.0013 | 0.2230 | 0.0984 | 4.14E-01 | 1.00E+00 |
| *DCC* | 50 | 18 | 50056896 | 50109971 | 0.0010 | 0.0019 | 0.2224 | 0.0984 | 2.50E-01 | 1.00E+00 |
| *DCC* | 50 | 18 | 50088019 | 50134134 | 0.0013 | 0.0018 | 0.2233 | 0.0985 | 1.13E-01 | 1.00E+00 |
| *DCC* | 50 | 18 | 50124500 | 50157653 | 0.0010 | 0.0015 | 0.2235 | 0.0985 | 1.16E-01 | 1.00E+00 |
| *DCC* | 50 | 18 | 50142425 | 50187597 | 0.0007 | 0.0016 | 0.2217 | 0.0984 | 2.73E-01 | 1.00E+00 |
| *DCC* | 50 | 18 | 50173450 | 50208962 | 0.0005 | 0.0013 | 0.2206 | 0.0984 | 2.83E-01 | 1.00E+00 |
| *DCC* | 50 | 18 | 50193561 | 50225314 | 0.0006 | 0.0012 | 0.2189 | 0.0984 | 1.90E-01 | 1.00E+00 |
| *DCC* | 50 | 18 | 50212069 | 50248759 | 0.0005 | 0.0011 | 0.2196 | 0.0985 | 2.65E-01 | 1.00E+00 |
| *DCC* | 50 | 18 | 50232913 | 50270259 | 0.0004 | 0.0011 | 0.2197 | 0.0985 | 2.76E-01 | 1.00E+00 |

| **Gene** | **W Size** | **Chr** | **Start_pos** | **End_pos** | **$h^2_R$** | **SE ($h^2_R$)** | **$h^2_C$** | **SE ($h^2_C$)** | **LRT ($h^2_R$) *P* Value** | **LRT ($h^2_R$) $P_{bonf}$** |
|---|---|---|---|---|---|---|---|---|---|---|
| *DCC* | 50 | 18 | 50253239 | 50307252 | 0.0004 | 0.0013 | 0.2207 | 0.0985 | 3.49E-01 | 1.00E+00 |
| *DCC* | 50 | 18 | 50297263 | 50336979 | 0.0000 | 0.0017 | 0.2273 | 0.0985 | 5.00E-01 | 1.00E+00 |
| *DCC* | 50 | 18 | 50310263 | 50354847 | 0.0000 | 0.0024 | 0.2276 | 0.0984 | 5.00E-01 | 1.00E+00 |
| *DCC* | 50 | 18 | 50348923 | 50366840 | 0.0019 | 0.0027 | 0.2126 | 0.0985 | 1.55E-01 | 1.00E+00 |
| *DCC* | 50 | 18 | 50357551 | 50400404 | 0.0054 | 0.0052 | 0.2062 | 0.0983 | 1.26E-02 | 5.29E-01 |
| *DCC* | 50 | 18 | 50371724 | 50424363 | 0.0034 | 0.0033 | 0.2063 | 0.0984 | 7.98E-03 | 3.35E-01 |
| *DCC* | 50 | 18 | 50412521 | 50446555 | 0.0021 | 0.0024 | 0.2080 | 0.0984 | 1.43E-02 | 6.01E-01 |
| *DCC* | 50 | 18 | 50434994 | 50472004 | 0.0024 | 0.0026 | 0.2067 | 0.0984 | 6.70E-03 | 2.81E-01 |
| *DCC* | 50 | 18 | 50454598 | 50514384 | 0.0024 | 0.0023 | 0.2080 | 0.0984 | 5.96E-03 | 2.50E-01 |
| *DCC* | 50 | 18 | 50484996 | 50544871 | 0.0031 | 0.0031 | 0.2117 | 0.0984 | 1.68E-02 | 7.06E-01 |
| *DCC* | 50 | 18 | 50527261 | 50589083 | 0.0053 | 0.0046 | 0.2078 | 0.0983 | 8.47E-03 | 3.56E-01 |
| *DCC* | 50 | 18 | 50555225 | 50623189 | 0.0033 | 0.0033 | 0.2045 | 0.0983 | 2.75E-03 | 1.16E-01 |
| *DCC* | 50 | 18 | 50609302 | 50642307 | 0.0042 | 0.0045 | 0.2038 | 0.0983 | 2.67E-03 | 1.12E-01 |
| *DCC* | 50 | 18 | 50631882 | 50683213 | 0.0039 | 0.0041 | 0.2044 | 0.0983 | 4.22E-03 | 1.77E-01 |
| *DCC* | 50 | 18 | 50661880 | 50714232 | 0.0025 | 0.0026 | 0.2065 | 0.0984 | 6.60E-03 | 2.77E-01 |
| *DCC* | 50 | 18 | 50691838 | 50747812 | 0.0025 | 0.0026 | 0.2068 | 0.0984 | 1.43E-02 | 6.01E-01 |
| *DCC* | 50 | 18 | 50728987 | 50810319 | 0.0037 | 0.0037 | 0.2054 | 0.0983 | 7.55E-03 | 3.17E-01 |
| *DCC* | 50 | 18 | 50769543 | 50835809 | 0.0057 | 0.0064 | 0.2063 | 0.0982 | 1.66E-02 | 6.97E-01 |
| *DCC* | 50 | 18 | 50819580 | 50870279 | 0.0017 | 0.0022 | 0.2084 | 0.0985 | 8.14E-02 | 1.00E+00 |
| *DCC* | 50 | 18 | 50851974 | 50906031 | 0.0024 | 0.0030 | 0.2055 | 0.0984 | 4.69E-02 | 1.00E+00 |
| *DCC* | 50 | 18 | 50887961 | 50932374 | 0.0028 | 0.0032 | 0.2053 | 0.0984 | 2.80E-02 | 1.00E+00 |
| *DCC* | 50 | 18 | 50918311 | 50972182 | 0.0041 | 0.0051 | 0.2069 | 0.0984 | 3.46E-02 | 1.00E+00 |
| *DCC* | 50 | 18 | 50941962 | 51053176 | 0.0029 | 0.0036 | 0.2057 | 0.0984 | 1.64E-02 | 6.89E-01 |
| *DCC* | 50 | 18 | 50994208 | 51071812 | 0.0026 | 0.0027 | 0.2057 | 0.0984 | 8.73E-03 | 3.67E-01 |
| *DCC* | 50 | 18 | 51063768 | 51071812 | 0.0000 | 0.0009 | 0.2235 | 0.0984 | 5.00E-01 | 1.00E+00 |
| *UNC5D* | 100 | 8 | 35073360 | 35173696 | 0.0040 | 0.0045 | 0.2409 | 0.0991 | 1.15E-02 | 5.75E-02 |
| *UNC5D* | 100 | 8 | 35162648 | 35273389 | 0.0030 | 0.0032 | 0.2406 | 0.0991 | 1.14E-02 | 5.70E-02 |
| *UNC5D* | 100 | 8 | 35247297 | 35397152 | 0.0031 | 0.0029 | 0.2426 | 0.0992 | 1.72E-02 | 8.60E-02 |
| *UNC5D* | 100 | 8 | 35362941 | 35493586 | 0.0000 | 0.0012 | 0.2509 | 0.0992 | 5.00E-01 | 1.00E+00 |
| *UNC5D* | 100 | 8 | 35467287 | 35645991 | 0.0000 | 0.0010 | 0.2507 | 0.0992 | 5.00E-01 | 1.00E+00 |
| *UNC5D* | 50 | 8 | 35073360 | 35143592 | 0.0040 | 0.0041 | 0.2125 | 0.0983 | 8.01E-03 | 1.04E-01 |
| *UNC5D* | 50 | 8 | 35125981 | 35162563 | 0.0038 | 0.0043 | 0.2107 | 0.0982 | 7.19E-03 | 9.35E-02 |
| *UNC5D* | 50 | 8 | 35151747 | 35179429 | 0.0046 | 0.0060 | 0.2109 | 0.0982 | 1.10E-02 | 1.43E-01 |
| *UNC5D* | 50 | 8 | 35167073 | 35218062 | 0.0029 | 0.0034 | 0.2109 | 0.0983 | 1.50E-02 | 1.95E-01 |

| **Gene** | **W Size** | **Chr** | **Start_pos** | **End_pos** | **$h^2_R$** | **SE ($h^2_R$)** | **$h^2_C$** | **SE ($h^2_C$)** | **LRT ($h^2_R$) *P* Value** | **LRT ($h^2_R$) $P_{bonf}$** |
|---|---|---|---|---|---|---|---|---|---|---|
| *UNC5D* | 50 | 8 | 35182128 | 35258054 | 0.0031 | 0.0031 | 0.2112 | 0.0983 | 7.03E-03 | 9.14E-02 |
| *UNC5D* | 50 | 8 | 35229398 | 35302656 | 0.0029 | 0.0030 | 0.2123 | 0.0983 | 5.33E-03 | 6.93E-02 |
| *UNC5D* | 50 | 8 | 35273524 | 35341127 | 0.0034 | 0.0031 | 0.2123 | 0.0983 | 7.59E-03 | 9.87E-02 |
| *UNC5D* | 50 | 8 | 35315188 | 35397152 | 0.0000 | 0.0008 | 0.2235 | 0.0984 | 5.00E-01 | 1.00E+00 |
| *UNC5D* | 50 | 8 | 35362941 | 35423794 | 0.0000 | 0.0011 | 0.2236 | 0.0984 | 5.00E-01 | 1.00E+00 |
| *UNC5D* | 50 | 8 | 35408064 | 35465022 | 0.0000 | 0.0009 | 0.2235 | 0.0984 | 5.00E-01 | 1.00E+00 |
| *UNC5D* | 50 | 8 | 35433083 | 35528279 | 0.0000 | 0.0006 | 0.2235 | 0.0984 | 5.00E-01 | 1.00E+00 |
| *UNC5D* | 50 | 8 | 35484224 | 35645991 | 0.0000 | 0.0012 | 0.2231 | 0.0984 | 5.00E-01 | 1.00E+00 |
| *UNC5D* | 50 | 8 | 35597155 | 35645991 | 0.0000 | 0.0008 | 0.2235 | 0.0984 | 5.00E-01 | 1.00E+00 |

W Size: window size. *P* (LRT): Nominal *P* value from LRT for $h_R^2$. $\boldsymbol{P}_{\mathbf{bonf}}$: Adjusted LRT *P* value using the Bonferroni method.

**Table S4A. SNP heritability (the heritability explained by all the imputed SNPs) for MDD in GS:SFHS.**

| **Sample** | **LRT ($h^2_{GWAS}$) *P* Value** | **$h^2_{GWAS}$** | **SE ($h^2_{GWAS}$)** |
|---|---|---|---|
| GS:SFHS | 4.92E-03 * | 0.251 | 0.099 |

* significant results.

**Table S4B. SNP heritability (the heritability explained by all the imputed SNPs) for MDD in combined set and each of the subsets in PGC:MDD.**

| **Group** | **LRT ($h^2_{GWAS}$) *P* value** | **$h^2_{GWAS}$** | **SE ($h^2_{GWAS}$)** |
|---|---|---|---|
| subset1 | 2.91E-06 * | 0.265 | 0.060 |
| subset2 | 0.00E+00 * | 0.467 | 0.052 |
| subset3 | 2.86E-09 * | 0.445 | 0.078 |
| combined | 0.00E+00 * | 0.285 | 0.022 |

* significant results.

**Table S5A. Gene-regional heritability attributable to genes in the pathway that had significant pathway-level regional heritability in GS:SFHS.**

| Pathway | Gene | LRT ($h^2_R$) *P* Value | $h^2_R$ | SE ($h^2_R$) | $h^2_C$ | SE ($h^2_C$) |
|---|---|---|---|---|---|---|
| NETRIN1 | *DCC* | 6.68E-03 * | 0.0041 | 0.0033 | 0.2332 | 0.0993 |
| NETRIN1 | *UNC5D* | 1.69E-02 * | 0.0033 | 0.0031 | 0.2417 | 0.0992 |
| NETRIN1 | *SIAH2* | 2.40E-02 * | 0.0024 | 0.0028 | 0.2509 | 0.0991 |
| NETRIN1 | *NEO1* | 7.64E-02 | 0.0015 | 0.0021 | 0.2452 | 0.0992 |
| NETRIN1 | *FYN* | 8.51E-02 | 0.0028 | 0.0030 | 0.2468 | 0.0992 |
| NETRIN1 | *EZR* | 9.86E-02 | 0.0012 | 0.0018 | 0.2442 | 0.0992 |
| NETRIN1 | *RAC1* | 1.80E-01 | 0.0010 | 0.0017 | 0.2490 | 0.0992 |
| NETRIN1 | *PLCG1* | 2.35E-01 | 0.0006 | 0.0013 | 0.2517 | 0.0993 |
| NETRIN1 | *RGMA* | 2.95E-01 | 0.0006 | 0.0016 | 0.2517 | 0.0993 |
| NETRIN1 | *RGMB* | 3.09E-01 | 0.0004 | 0.0011 | 0.2513 | 0.0993 |
| NETRIN1 | *MYO10* | 3.11E-01 | 0.0013 | 0.0029 | 0.2514 | 0.0992 |
| NETRIN1 | *TRPC6* | 3.49E-01 | 0.0004 | 0.0013 | 0.2517 | 0.0993 |
| NETRIN1 | *NTN4* | 3.74E-01 | 0.0004 | 0.0016 | 0.2522 | 0.0993 |
| NETRIN1 | *DOCK1* | 4.42E-01 | 0.0003 | 0.0020 | 0.2512 | 0.0992 |
| NETRIN1 | *ABLIM1* | 5.00E-01 | 0.0000 | 0.0015 | 0.2513 | 0.0993 |
| NETRIN1 | *ABLIM3* | 5.00E-01 | 0.0000 | 0.0017 | 0.2525 | 0.0993 |
| NETRIN1 | *CDC42* | 5.00E-01 | 0.0000 | 0.0020 | 0.2509 | 0.0993 |
| NETRIN1 | *NCK1* | 5.00E-01 | 0.0000 | 0.0008 | 0.2511 | 0.0993 |
| NETRIN1 | *NTN1* | 5.00E-01 | 0.0000 | 0.0015 | 0.2515 | 0.0993 |
| NETRIN1 | *PITPNA* | 5.00E-01 | 0.0000 | 0.0018 | 0.2523 | 0.0993 |
| NETRIN1 | *PRKCQ* | 5.00E-01 | 0.0000 | 0.0018 | 0.2508 | 0.0993 |
| NETRIN1 | *PTK2* | 5.00E-01 | 0.0000 | 0.0084 | 0.2341 | 0.0970 |
| NETRIN1 | *ROBO1* | 5.00E-01 | 0.0000 | 0.0022 | 0.2524 | 0.0993 |
| NETRIN1 | *SLIT1* | 5.00E-01 | 0.0000 | 0.0016 | 0.2505 | 0.0992 |
| NETRIN1 | *SLIT2* | 5.00E-01 | 0.0000 | 0.0026 | 0.2492 | 0.0992 |
| NETRIN1 | *SLIT3* | 5.00E-01 | 0.0000 | 0.0031 | 0.2512 | 0.0992 |
| NETRIN1 | *SRC* | 5.00E-01 | 0.0000 | 0.0016 | 0.2480 | 0.0993 |
| NETRIN1 | *TRIO* | 5.00E-01 | 0.0000 | 0.0020 | 0.2535 | 0.0993 |
| NETRIN1 | *TRPC3* | 5.00E-01 | 0.0000 | 0.0011 | 0.2516 | 0.0993 |
| NETRIN1 | *TRPC4* | 5.00E-01 | 0.0000 | 0.0018 | 0.2522 | 0.0993 |

| **Pathway** | **Gene** | **LRT ($h^2_R$) *P* Value** | **$h^2_R$** | **SE ($h^2_R$)** | **$h^2_C$** | **SE ($h^2_C$)** |
|---|---|---|---|---|---|---|
| NETRIN1 | *TRPC7* | 5.00E-01 | 0.0000 | 0.0010 | 0.2514 | 0.0993 |
| NETRIN1 | *UNC5A* | 5.00E-01 | 0.0000 | 0.0010 | 0.2514 | 0.0993 |
| NETRIN1 | *UNC5B* | 5.00E-01 | 0.0000 | 0.0026 | 0.2487 | 0.0992 |
| NETRIN1 | *UNC5C* | 5.00E-01 | 0.0000 | 0.0031 | 0.2510 | 0.0992 |
| NETRIN1 | *WASL* | 5.00E-01 | 0.0000 | 0.0013 | 0.2539 | 0.0993 |
| MTORC1 | *RPTOR* | 0.01182 * | 0.0042 | 0.0035 | 0.2424 | 0.0990 |
| MTORC1 | *RHEB* | 0.0508 | 0.0012 | 0.0017 | 0.2532 | 0.0993 |
| MTORC1 | *MTOR* | 0.1434 | 0.0008 | 0.0014 | 0.2499 | 0.0992 |
| MTORC1 | *EEF2K* | 0.5 | 0.0000 | 0.0008 | 0.2515 | 0.0993 |
| MTORC1 | *EIF4B* | 0.5 | 0.0000 | 0.0006 | 0.2514 | 0.0993 |
| MTORC1 | *EIF4EBP1* | 0.5 | 0.0000 | 0.0011 | 0.2521 | 0.0993 |
| MTORC1 | *EIF4E* | 0.5 | 0.0000 | 0.0027 | 0.2462 | 0.0991 |
| MTORC1 | *RPS6KB1* | 0.5 | 0.0000 | 0.0016 | 0.2515 | 0.0992 |

* significant results.

**Table S5B. Gene-regional heritability attributable to genes in the pathway that had significant pathway-level regional heritability (the role of second messengers in NETRIN1 signaling pathway) in PGC:MDD.**

| Group | Gene | LRT ($h^2_R$) *P* Value | $h^2_R$ | SE ($h^2_R$) | $h^2_C$ | SE ($h^2_C$) |
|---|---|---|---|---|---|---|
| Subset1 | *DCC* | 1.99E-02 * | 0.0031 | 0.0024 | 0.2650 | 0.0596 |
| Subset1 | *NTN1* | 3.29E-01 | 0.0007 | 0.0016 | 0.2644 | 0.0597 |
| Subset1 | *PITPNA* | 5.00E-01 | 0.0000 | 0.0006 | 0.2650 | 0.0597 |
| Subset1 | *PLCG1* | 8.97E-02 | 0.0014 | 0.0017 | 0.2634 | 0.0597 |
| Subset1 | *TRPC3* | 5.00E-01 | 0.0000 | 0.0008 | 0.2641 | 0.0597 |
| Subset1 | *TRPC4* | 1.48E-01 | 0.0009 | 0.0013 | 0.2606 | 0.0597 |
| Subset1 | *TRPC6* | 4.73E-01 | 0.0001 | 0.0008 | 0.2648 | 0.0597 |
| Subset1 | *TRPC7* | 2.68E-01 | 0.0003 | 0.0008 | 0.2637 | 0.0597 |
| Subset2 | *DCC* | 3.99E-01 | 0.0002 | 0.0009 | 0.4667 | 0.0525 |
| Subset2 | *NTN1* | 5.00E-01 | 0.0000 | 0.0013 | 0.4669 | 0.0525 |
| Subset2 | *PITPNA* | 2.66E-01 | 0.0004 | 0.0009 | 0.4661 | 0.0525 |
| Subset2 | *PLCG1* | 2.14E-01 | 0.0004 | 0.0008 | 0.4660 | 0.0525 |
| Subset2 | *TRPC3* | 5.00E-01 | 0.0000 | 0.0011 | 0.4668 | 0.0525 |
| Subset2 | *TRPC4* | 2.46E-01 | 0.0006 | 0.0011 | 0.4664 | 0.0525 |
| Subset2 | *TRPC6* | 5.00E-01 | 0.0000 | 0.0005 | 0.4668 | 0.0525 |
| Subset2 | *TRPC7* | 1.21E-01 | 0.0007 | 0.0010 | 0.4651 | 0.0525 |
| Subset3 | *DCC* | 3.95E-01 | 0.0003 | 0.0014 | 0.4443 | 0.0781 |
| Subset3 | *NTN1* | 3.75E-01 | 0.0004 | 0.0014 | 0.4439 | 0.0781 |
| Subset3 | *PITPNA* | 2.69E-01 | 0.0005 | 0.0011 | 0.4409 | 0.0782 |
| Subset3 | *PLCG1* | 3.81E-01 | 0.0006 | 0.0015 | 0.4451 | 0.0781 |
| Subset3 | *TRPC3* | 1.18E-02 * | 0.0055 | 0.0047 | 0.4476 | 0.0778 |
| Subset3 | *TRPC4* | 5.00E-01 | 0.0000 | 0.0017 | 0.4445 | 0.0781 |
| Subset3 | *TRPC6* | 5.00E-01 | 0.0000 | 0.0010 | 0.4450 | 0.0781 |
| Subset3 | *TRPC7* | 5.00E-01 | 0.0000 | 0.0007 | 0.4444 | 0.0781 |
| Combined | *DCC* | 2.44E-01 | 0.0002 | 0.0005 | 0.2844 | 0.0220 |
| Combined | *NTN1* | 5.00E-01 | 0.0000 | 0.0005 | 0.2847 | 0.0221 |
| Combined | *PITPNA* | 3.32E-02 * | 0.0006 | 0.0007 | 0.2839 | 0.0221 |
| Combined | *PLCG1* | 7.36E-03 * | 0.0010 | 0.0010 | 0.2836 | 0.0220 |

| **Group** | **Gene** | **LRT ($h^2_R$) *P* Value** | **$h^2_R$** | **SE ($h^2_R$)** | **$h^2_C$** | **SE ($h^2_C$)** |
|---|---|---|---|---|---|---|
| Combined | *TRPC3* | 9.02E-02 | 0.0020 | 0.0018 | 0.2844 | 0.0220 |
| Combined | *TRPC4* | 5.00E-01 | 0.0000 | 0.0007 | 0.2851 | 0.0220 |
| Combined | *TRPC6* | 5.00E-01 | 0.0000 | 0.0009 | 0.2829 | 0.0220 |
| Combined | *TRPC7* | 1.86E-01 | 0.0002 | 0.0003 | 0.2844 | 0.0221 |

* significant results.

**Table S6A. RHM result for *DCC* and *UNC5D* in GS:SFHS using a window of size 200 SNPs.**

| Gene | Chr | Start_pos | End_pos | $h^2_R$ | se($h^2_R$) | $h^2_C$ | SE ($h^2_C$) | LRT ($h^2_R$) *P* Value | LRT ($h^2_R$) $P_{bonf}$ |
|---|---|---|---|---|---|---|---|---|---|
| *DCC* | 18 | 49847408 | 50002997 | 0.0000 | 0.0025 | 0.2494 | 0.0992 | 5.00E-01 | 1.00E+00 |
| *DCC* | 18 | 49952593 | 50134134 | 0.0016 | 0.0024 | 0.2496 | 0.0993 | 1.60E-01 | 1.00E+00 |
| *DCC* | 18 | 50088019 | 50248759 | 0.0011 | 0.0017 | 0.2468 | 0.0993 | 1.48E-01 | 1.00E+00 |
| *DCC* | 18 | 50212069 | 50366840 | 0.0005 | 0.0015 | 0.2473 | 0.0994 | 3.38E-01 | 1.00E+00 |
| *DCC* | 18 | 50348923 | 50514384 | 0.0030 | 0.0029 | 0.2342 | 0.0993 | 9.27E-03 | 8.34E-02 |
| *DCC* | 18 | 50454598 | 50683213 | 0.0036 | 0.0031 | 0.2317 | 0.0992 | 2.39E-03 | 2.15E-02 * |
| *DCC* | 18 | 50631882 | 50870279 | 0.0027 | 0.0027 | 0.2328 | 0.0993 | 1.03E-02 | 9.27E-02 |
| *DCC* | 18 | 50819580 | 51071812 | 0.0033 | 0.0037 | 0.2327 | 0.0993 | 2.37E-02 | 2.13E-01 |
| *DCC* | 18 | 50994208 | 51071812 | 0.0026 | 0.0027 | 0.2335 | 0.0993 | 9.01E-03 | 8.11E-02 |
| *UNC5D* | 8 | 35073360 | 35302656 | 0.0031 | 0.0033 | 0.2407 | 0.0991 | 9.38E-03 | 2.81E-02 * |
| *UNC5D* | 8 | 35229398 | 35528279 | 0.0038 | 0.0034 | 0.2428 | 0.0992 | 3.07E-02 | 9.21E-02 |
| *UNC5D* | 8 | 35433083 | 35645991 | 0.0000 | 0.0007 | 0.2510 | 0.0992 | 4.95E-01 | 1.00E+00 |

LRT ($h^2_R$) $P_{bonf}$: adjusted *P* value by Bonferroni correction ($N_{Bonf\_for\_DCC}$ = 9, $N_{Bonf\_for\_UNC5D}$ = 3).
* significant results.

**Table S6B. RHM result for *DCC* in subset1 in PGC:MDD using a window of size 100 SNPs.**

| Gene | Chr | Start_pos | End_pos | $h^2_R$ | SE ($h^2_R$) | $h^2_C$ | SE ($h^2_C$) | LRT ($h^2_R$) *P* Value | LRT ($h^2_R$) $P_{bonf}$ |
|---|---|---|---|---|---|---|---|---|---|
| *DCC* | 18 | 49847072 | 50010438 | 0.0000 | 0.0010 | 0.2646 | 0.0597 | 5.00E-01 | 1.00E+00 |
| *DCC* | 18 | 49966318 | 50178146 | 0.0000 | 0.0012 | 0.2636 | 0.0597 | 5.00E-01 | 1.00E+00 |
| *DCC* | 18 | 50137957 | 50310263 | 0.0022 | 0.0020 | 0.2653 | 0.0596 | 2.58E-02 | 2.06E-01 |
| *DCC* | 18 | 50262963 | 50475010 | 0.0032 | 0.0028 | 0.2665 | 0.0596 | 3.66E-02 | 2.93E-01 |
| *DCC* | 18 | 50430275 | 50670111 | 0.0019 | 0.0021 | 0.2669 | 0.0597 | 4.72E-02 | 3.78E-01 |
| *DCC* | 18 | 50621094 | 50861056 | 0.0021 | 0.0023 | 0.2672 | 0.0597 | 4.87E-02 | 3.90E-01 |
| *DCC* | 18 | 50815036 | 51065247 | 0.0022 | 0.0026 | 0.2662 | 0.0597 | 1.43E-01 | 1.00E+00 |
| *DCC* | 18 | 51024806 | 51065247 | 0.0000 | 0.0013 | 0.2626 | 0.0596 | 5.00E-01 | 1.00E+00 |

LRT ($h^2_R$) $P_{bonf}$: adjusted *P* value by Bonferroni correction ($N_{Bonf\_for\_DCC}$ = 8).

**Table S7. Comparison of the variance explained by the fixed effect from NETRIN1 signaling PRS and whole genome PRS, and the *t* test result of the effect in logistic regression.**

| LD Clumping | Source of SNPs | *P* Cutoff | *P* Value | $R^2$ | $N_{snps}$ |
|---|---|---|---|---|---|
| no | NETRIN1 signaling | 0.01 | 0.179 | 0.00048 | 17 |
| no | NETRIN1 signaling | 0.05 | 0.029 * | 0.00128 | 104 |
| no | NETRIN1 signaling | 0.10 | 0.017 * | 0.00153 | 174 |
| no | NETRIN1 signaling | 0.20 | 0.004 * | 0.00216 | 328 |
| no | NETRIN1 signaling | 0.50 | 0.006 * | 0.00202 | 721 |
| no | NETRIN1 signaling | 1.00 | 0.010 * | 0.00176 | 1562 |
| yes | NETRIN1 signaling | 0.01 | 0.181 | 0.00048 | 12 |
| yes | NETRIN1 signaling | 0.05 | 0.034 * | 0.00120 | 43 |
| yes | NETRIN1 signaling | 0.10 | 0.083 | 0.00080 | 71 |
| yes | NETRIN1 signaling | 0.20 | 0.059 | 0.00096 | 142 |
| yes | NETRIN1 signaling | 0.50 | 0.035 * | 0.00118 | 291 |
| yes | NETRIN1 signaling | 1.00 | 0.083 | 0.00106 | 489 |
| no | whole genome | 0.01 | 0.235 | 0.00038 | 5469 |
| no | whole genome | 0.05 | 0.032 * | 0.00122 | 24443 |
| no | whole genome | 0.10 | 0.010 * | 0.00179 | 47026 |
| no | whole genome | 0.20 | 0.006 * | 0.00198 | 90620 |
| no | whole genome | 0.50 | 0.009 * | 0.00183 | 215698 |
| no | whole genome | 1.00 | 0.007 * | 0.00193 | 419635 |
| yes | whole genome | 0.01 | 0.661 | 0.00005 | 2819 |
| yes | whole genome | 0.05 | 0.201 | 0.00044 | 11170 |
| yes | whole genome | 0.10 | 0.046 * | 0.00106 | 20212 |
| yes | whole genome | 0.20 | 0.011 * | 0.00172 | 35909 |
| yes | whole genome | 0.50 | 0.031 * | 0.00124 | 73298 |
| yes | whole genome | 1.00 | 0.014 * | 0.00161 | 110590 |

*P* Cutoff: GWAS *P* value cutoff for SNPs used in creating PRS. *P* Value: *t* test result for PRS as fixed effect. $R^2$ (Nagelkerke's $R^2$): the variance explained by PRS on the observed scale. $N_{snps}$: number of SNPs used in creating PRS. * significant results.

**Table S8. Permutation result for NETRIN1 PRS (without LD clumping) in logistic regression.**

| PRS | *P* Cutoff | *P* Value (P) | *P* Value ($R^2$) |
|---|---|---|---|
| NETRIN1 signaling | 0.01 | 0.212 | 0.215 |
| NETRIN1 signaling | 0.05 | 0.037 * | 0.037 * |
| NETRIN1 signaling | 0.10 | 0.02 * | 0.021 * |
| NETRIN1 signaling | 0.20 | 0.006 * | 0.008 * |
| NETRIN1 signaling | 0.50 | 0.008 * | 0.008 * |
| NETRIN1 signaling | 1.00 | 0.011 * | 0.012 * |

*P* Value (P): permutation *P* value in terms of *P* value from *t* test for PRS as fixed effect. *P* Value ($R^2$): permutation *P* value in terms of the variance explained by PRS. * significant results.

**Table S9. Comparison of the variance explained by the random effect from the NETRIN1 signaling PRS and whole genome PRS in LMM.**

| Var1: PRS-bin Relationship Matrix | | | | | | | Var2: GRM | |
|---|---|---|---|---|---|---|---|---|
| **PRS SNP** | **LD Clumping** | $P_{cutoff}$ | **Bin** | **LRT ($h^2_{PRS}$) *P* Value** | $h^2_{PRS}$ | **SE ($h^2_{PRS}$)** | $h^2_g$ | **SE ($h^2_g$)** |
| NETRIN1 signaling | No | 0.2 | 10 | 5.68E-02 | 0.0071 | 0.0095 | 0.2405 | 0.0989 |
| NETRIN1 signaling | No | 0.2 | 20 | 2.55E-02 * | 0.0099 | 0.0118 | 0.2390 | 0.0988 |
| NETRIN1 signaling | No | 0.2 | 50 | 1.81E-02 * | 0.0121 | 0.0139 | 0.2381 | 0.0987 |
| NETRIN1 signaling | No | 0.2 | 200 | 1.86E-02 * | 0.0115 | 0.0133 | 0.2380 | 0.0987 |
| NETRIN1 signaling | No | 0.2 | 300 | 1.83E-02 * | 0.0115 | 0.0133 | 0.2379 | 0.0987 |
| NETRIN1 signaling | No | 0.2 | 500 | 1.89E-02 * | 0.0114 | 0.0132 | 0.2380 | 0.0987 |
| NETRIN1 signaling | No | 0.2 | 1000 | 1.93E-02 * | 0.0113 | 0.0131 | 0.2380 | 0.0987 |
| NETRIN1 signaling | No | 0.2 | 6000 | 1.94E-02 * | 0.0113 | 0.0131 | 0.2380 | 0.0987 |
| NETRIN1 signaling | No | 0.5 | 10 | 4.53E-02 * | 0.0098 | 0.0128 | 0.2403 | 0.0988 |
| NETRIN1 signaling | No | 0.5 | 20 | 1.65E-02 * | 0.0138 | 0.0161 | 0.2376 | 0.0986 |
| NETRIN1 signaling | No | 0.5 | 50 | 1.30E-02 * | 0.0170 | 0.0199 | 0.2372 | 0.0985 |
| NETRIN1 signaling | No | 0.5 | 100 | 1.47E-02 * | 0.0154 | 0.0180 | 0.2368 | 0.0986 |
| NETRIN1 signaling | No | 0.5 | 200 | 1.44E-02 * | 0.0153 | 0.0178 | 0.2367 | 0.0986 |
| NETRIN1 signaling | No | 0.5 | 300 | 1.40E-02 * | 0.0149 | 0.0175 | 0.2368 | 0.0986 |
| NETRIN1 signaling | No | 0.5 | 500 | 1.47E-02 * | 0.0149 | 0.0175 | 0.2368 | 0.0986 |
| NETRIN1 signaling | No | 0.5 | 6000 | 1.45E-02 * | 0.0150 | 0.0176 | 0.2368 | 0.0986 |
| NETRIN1 signaling | No | 1 | 10 | 4.60E-02 * | 0.0083 | 0.0109 | 0.2404 | 0.0989 |
| NETRIN1 signaling | No | 1 | 20 | 4.90E-02 * | 0.0077 | 0.0101 | 0.2395 | 0.0990 |
| NETRIN1 signaling | No | 1 | 50 | 3.46E-02 * | 0.0098 | 0.0126 | 0.2377 | 0.0989 |
| NETRIN1 signaling | No | 1 | 200 | 3.85E-02 * | 0.0095 | 0.0125 | 0.2382 | 0.0989 |
| NETRIN1 signaling | No | 1 | 300 | 3.92E-02 * | 0.0094 | 0.0124 | 0.2382 | 0.0989 |
| NETRIN1 signaling | No | 1 | 500 | 3.83E-02 * | 0.0096 | 0.0126 | 0.2382 | 0.0989 |
| NETRIN1 signaling | No | 1 | 1000 | 3.84E-02 * | 0.0096 | 0.0125 | 0.2382 | 0.0989 |
| NETRIN1 signaling | No | 1 | 6000 | 3.84E-02 * | 0.0095 | 0.0125 | 0.2382 | 0.0989 |
| NETRIN1 signaling | yes | 0.2 | 10 | 0.1421 | 0.0059 | 0.0101 | 0.2477 | 0.0990 |
| NETRIN1 signaling | yes | 0.2 | 20 | 0.1129 | 0.0073 | 0.0115 | 0.2463 | 0.0989 |
| NETRIN1 signaling | yes | 0.2 | 50 | 0.1481 | 0.0059 | 0.0105 | 0.2470 | 0.0990 |
| NETRIN1 signaling | yes | 0.2 | 200 | 0.1375 | 0.0063 | 0.0109 | 0.2468 | 0.0990 |
| NETRIN1 signaling | yes | 0.2 | 300 | 0.1262 | 0.0070 | 0.0117 | 0.2466 | 0.0989 |
| NETRIN1 signaling | yes | 0.2 | 500 | 0.1282 | 0.0068 | 0.0114 | 0.2467 | 0.0989 |
| NETRIN1 signaling | yes | 0.2 | 1000 | 0.1283 | 0.0068 | 0.0114 | 0.2467 | 0.0989 |
| NETRIN1 signaling | yes | 0.2 | 6000 | 0.1273 | 0.0069 | 0.0115 | 0.2467 | 0.0989 |
| NETRIN1 signaling | yes | 0.5 | 10 | 0.06559 | 0.0104 | 0.0144 | 0.2434 | 0.0988 |
| NETRIN1 signaling | yes | 0.5 | 20 | 0.05885 | 0.0101 | 0.0138 | 0.2448 | 0.0988 |
| NETRIN1 signaling | yes | 0.5 | 50 | 0.05993 | 0.0109 | 0.0152 | 0.2453 | 0.0987 |
| NETRIN1 signaling | yes | 0.5 | 200 | 0.06119 | 0.0105 | 0.0145 | 0.2451 | 0.0988 |
| NETRIN1 signaling | yes | 0.5 | 300 | 0.06163 | 0.0105 | 0.0146 | 0.2451 | 0.0988 |
| NETRIN1 signaling | yes | 0.5 | 500 | 0.06139 | 0.0104 | 0.0144 | 0.2451 | 0.0988 |
| NETRIN1 signaling | yes | 0.5 | 1000 | 0.06069 | 0.0107 | 0.0147 | 0.2450 | 0.0987 |
| NETRIN1 signaling | yes | 0.5 | 6000 | 0.06064 | 0.0107 | 0.0148 | 0.2450 | 0.0987 |
| NETRIN1 signaling | yes | 1 | 10 | 0.0613 | 0.0093 | 0.0129 | 0.2462 | 0.0988 |
| NETRIN1 signaling | yes | 1 | 20 | 0.09924 | 0.0093 | 0.0144 | 0.2459 | 0.0988 |
| NETRIN1 signaling | yes | 1 | 50 | 0.1266 | 0.0069 | 0.0115 | 0.2469 | 0.0989 |
| NETRIN1 signaling | yes | 1 | 200 | 0.09712 | 0.0085 | 0.0132 | 0.2462 | 0.0989 |
| NETRIN1 signaling | yes | 1 | 300 | 0.1003 | 0.0082 | 0.0128 | 0.2463 | 0.0989 |
| NETRIN1 signaling | yes | 1 | 500 | 0.09842 | 0.0084 | 0.0132 | 0.2462 | 0.0989 |
| NETRIN1 signaling | yes | 1 | 1000 | 0.0985 | 0.0084 | 0.0131 | 0.2462 | 0.0989 |
| NETRIN1 signaling | yes | 1 | 6000 | 0.09829 | 0.0084 | 0.0131 | 0.2462 | 0.0989 |

| Var1: PRS-bin Relationship Matrix | | | | | | | Var2: GRM | |
|---|---|---|---|---|---|---|---|---|
| **PRS SNP** | **LD Clumping** | $P_{cutoff}$ | **Bin** | **LRT ($h^2_{PRS}$) *P* Value** | $h^2_{PRS}$ | **SE ($h^2_{PRS}$)** | $h^2_g$ | **SE ($h^2_g$)** |
| whole genome | No | 0.2 | 10 | 0.3289 | 0.0022 | 0.0071 | 0.2485 | 0.0992 |
| whole genome | No | 0.2 | 20 | 0.2532 | 0.0056 | 0.0121 | 0.2465 | 0.0991 |
| whole genome | No | 0.2 | 50 | 0.172 | 0.0078 | 0.0137 | 0.2450 | 0.0990 |
| whole genome | No | 0.2 | 200 | 0.1643 | 0.0086 | 0.0148 | 0.2448 | 0.0989 |
| whole genome | No | 0.2 | 300 | 0.1589 | 0.0090 | 0.0152 | 0.2446 | 0.0989 |
| whole genome | No | 0.2 | 500 | 0.1582 | 0.0092 | 0.0154 | 0.2445 | 0.0989 |
| whole genome | No | 0.2 | 1000 | 0.1569 | 0.0093 | 0.0155 | 0.2445 | 0.0989 |
| whole genome | No | 0.2 | 6000 | 0.1578 | 0.0092 | 0.0154 | 0.2446 | 0.0989 |
| whole genome | No | 0.5 | 10 | 0.2801 | 0.0029 | 0.0076 | 0.2474 | 0.0992 |
| whole genome | No | 0.5 | 20 | 0.3219 | 0.0022 | 0.0069 | 0.2479 | 0.0992 |
| whole genome | No | 0.5 | 50 | 0.1816 | 0.0058 | 0.0110 | 0.2449 | 0.0990 |
| whole genome | No | 0.5 | 200 | 0.2092 | 0.0049 | 0.0099 | 0.2456 | 0.0991 |
| whole genome | No | 0.5 | 300 | 0.2061 | 0.0049 | 0.0099 | 0.2455 | 0.0991 |
| whole genome | No | 0.5 | 500 | 0.2072 | 0.0049 | 0.0099 | 0.2455 | 0.0991 |
| whole genome | No | 0.5 | 1000 | 0.2063 | 0.0049 | 0.0099 | 0.2455 | 0.0991 |
| whole genome | No | 0.5 | 6000 | 0.2081 | 0.0048 | 0.0098 | 0.2455 | 0.0991 |
| whole genome | No | 1 | 10 | 0.2783 | 0.0030 | 0.0075 | 0.2463 | 0.0992 |
| whole genome | No | 1 | 20 | 0.2272 | 0.0036 | 0.0077 | 0.2458 | 0.0992 |
| whole genome | No | 1 | 50 | 0.1868 | 0.0049 | 0.0094 | 0.2450 | 0.0991 |
| whole genome | No | 1 | 200 | 0.2033 | 0.0044 | 0.0088 | 0.2455 | 0.0991 |
| whole genome | No | 1 | 300 | 0.2029 | 0.0044 | 0.0089 | 0.2456 | 0.0991 |
| whole genome | No | 1 | 500 | 0.2016 | 0.0045 | 0.0089 | 0.2455 | 0.0991 |
| whole genome | No | 1 | 1000 | 0.2007 | 0.0045 | 0.0089 | 0.2455 | 0.0991 |
| whole genome | No | 1 | 6000 | 0.2005 | 0.0045 | 0.0089 | 0.2455 | 0.0991 |
| whole genome | Yes | 0.2 | 10 | 2.84E-02 * | 0.0247 | 0.0279 | 0.2394 | 0.0981 |
| whole genome | Yes | 0.2 | 20 | 3.16E-02 * | 0.0202 | 0.0237 | 0.2407 | 0.0983 |
| whole genome | Yes | 0.2 | 50 | 4.89E-02 * | 0.0137 | 0.0185 | 0.2423 | 0.0986 |
| whole genome | Yes | 0.2 | 200 | 5.46E-02 | 0.0120 | 0.0165 | 0.2424 | 0.0987 |
| whole genome | Yes | 0.2 | 300 | 5.60E-02 | 0.0116 | 0.0160 | 0.2425 | 0.0987 |
| whole genome | Yes | 0.2 | 500 | 5.66E-02 | 0.0116 | 0.0161 | 0.2425 | 0.0987 |
| whole genome | Yes | 0.2 | 1000 | 5.59E-02 | 0.0117 | 0.0162 | 0.2425 | 0.0987 |
| whole genome | Yes | 0.2 | 6000 | 5.54E-02 | 0.0117 | 0.0162 | 0.2425 | 0.0987 |
| whole genome | Yes | 0.5 | 10 | 9.03E-02 | 0.0083 | 0.0122 | 0.2430 | 0.0989 |
| whole genome | Yes | 0.5 | 20 | 1.22E-01 | 0.0064 | 0.0104 | 0.2439 | 0.0990 |
| whole genome | Yes | 0.5 | 50 | 1.52E-01 | 0.0053 | 0.0094 | 0.2452 | 0.0990 |
| whole genome | Yes | 0.5 | 200 | 1.40E-01 | 0.0057 | 0.0098 | 0.2450 | 0.0990 |
| whole genome | Yes | 0.5 | 300 | 1.43E-01 | 0.0055 | 0.0096 | 0.2451 | 0.0990 |
| whole genome | Yes | 0.5 | 500 | 1.41E-01 | 0.0056 | 0.0097 | 0.2450 | 0.0990 |
| whole genome | Yes | 0.5 | 1000 | 1.40E-01 | 0.0056 | 0.0098 | 0.2450 | 0.0990 |
| whole genome | Yes | 0.5 | 6000 | 1.40E-01 | 0.0056 | 0.0097 | 0.2450 | 0.0990 |
| whole genome | Yes | 1 | 10 | 8.87E-02 | 0.0091 | 0.0131 | 0.2427 | 0.0988 |
| whole genome | Yes | 1 | 20 | 1.18E-01 | 0.0064 | 0.0102 | 0.2437 | 0.0990 |
| whole genome | Yes | 1 | 50 | 6.43E-02 | 0.0092 | 0.0129 | 0.2421 | 0.0988 |
| whole genome | Yes | 1 | 200 | 6.85E-02 | 0.0092 | 0.0132 | 0.2423 | 0.0988 |
| whole genome | Yes | 1 | 300 | 7.10E-02 | 0.0090 | 0.0130 | 0.2425 | 0.0988 |
| whole genome | Yes | 1 | 500 | 7.03E-02 | 0.0090 | 0.0129 | 0.2424 | 0.0988 |
| whole genome | Yes | 1 | 1000 | 7.03E-02 | 0.0090 | 0.0129 | 0.2424 | 0.0988 |
| whole genome | Yes | 1 | 6000 | 7.00E-02 | 0.0090 | 0.0129 | 0.2424 | 0.0988 |

$P_{cutoff}$: GWAS *P* value cutoff for GWAS SNPs used in creating PRS. $h^2_{PRS}$: phenotypic variance explained by the PRS-bin relationship matrix. $h^2_g$: phenotypic variance explained by whole genome SNP GRM. $P$ (LRT): Nominal $P$ value from LRT for $h^2_{PRS}$. * significant results.

**Table S10. Phenotypic variance attributable to the three variance components by jointly fitting NETRIN1 signaling PRS-bin relationship matrix, whole genome PRS-bin relationship matrix and genomic relationship matrix in LMM.** This was to explore whether PRS from the NETRIN1 pathway explained additional variance when the genetic effect of the whole-genome PRS was taken into account. For the whole genome PRS-bin relationship matrix, a PRS created with LD clumping and with GWAS $P_{cutoff}$ = 0.2 and bin = 10 was adopted in this analysis as the PRS created at this threshold explained the highest proportion of variance (Table S9), which provides the largest variance component from the whole-genome PRS for the variance from NETRIN1 PRS-bin relationship matrix to compete against when jointly fitted.

| Var1: NETRIN1-PGS-bin Relationship Matrix | | | | | | Var2: Whole-genome-PGS-bin Relationship Matrix | | | | | Var3: GRM | |
|---|---|---|---|---|---|---|---|---|---|---|---|---|
| **PRS SNP** | $P_{cutoff}$ | **Bin** | **LRT ($h^2_{PRS}$) *P* Value** | $h^2_{PRS}$ | **SE ($h^2_{PRS}$)** | **PRS SNP** | $P_{cutoff}$ | **Bin** | $h^2_{PRS}$ | **SE ($h^2_{PRS}$)** | $h^2_g$ | **SE ($h^2_g$)** |
| NETRIN1 signaling | 0.2 | 10 | 7.19E-02 | 0.0064 | 0.0090 | whole genome | 0.2 | 10 | 0.0231 | 0.0267 | 0.2303 | 0.0979 |
| NETRIN1 signaling | 0.2 | 20 | 3.36E-02 * | 0.0091 | 0.0113 | whole genome | 0.2 | 10 | 0.0225 | 0.0262 | 0.2290 | 0.0978 |
| NETRIN1 signaling | 0.2 | 50 | 2.42E-02 * | 0.0112 | 0.0133 | whole genome | 0.2 | 10 | 0.0223 | 0.0261 | 0.2281 | 0.0977 |
| NETRIN1 signaling | 0.2 | 200 | 2.47E-02 * | 0.0107 | 0.0127 | whole genome | 0.2 | 10 | 0.0223 | 0.0261 | 0.2280 | 0.0977 |
| NETRIN1 signaling | 0.2 | 300 | 2.43E-02 * | 0.0107 | 0.0127 | whole genome | 0.2 | 10 | 0.0223 | 0.0261 | 0.2280 | 0.0977 |
| NETRIN1 signaling | 0.2 | 500 | 2.51E-02 * | 0.0106 | 0.0127 | whole genome | 0.2 | 10 | 0.0223 | 0.0261 | 0.2281 | 0.0977 |
| NETRIN1 signaling | 0.2 | 1000 | 2.56E-02 * | 0.0104 | 0.0125 | whole genome | 0.2 | 10 | 0.0223 | 0.0261 | 0.2281 | 0.0977 |
| NETRIN1 signaling | 0.2 | 6000 | 2.57E-02 * | 0.0104 | 0.0125 | whole genome | 0.2 | 10 | 0.0223 | 0.0261 | 0.2281 | 0.0977 |
| NETRIN1 signaling | 0.5 | 10 | 5.68E-02 | 0.0089 | 0.0122 | whole genome | 0.2 | 10 | 0.0231 | 0.0267 | 0.2301 | 0.0978 |
| NETRIN1 signaling | 0.5 | 20 | 2.15E-02 * | 0.0129 | 0.0155 | whole genome | 0.2 | 10 | 0.0226 | 0.0263 | 0.2276 | 0.0976 |
| NETRIN1 signaling | 0.5 | 50 | 1.74E-02 * | 0.0160 | 0.0192 | whole genome | 0.2 | 10 | 0.0224 | 0.0262 | 0.2273 | 0.0975 |
| NETRIN1 signaling | 0.5 | 200 | 1.89E-02 * | 0.0145 | 0.0175 | whole genome | 0.2 | 10 | 0.0225 | 0.0263 | 0.2268 | 0.0976 |
| NETRIN1 signaling | 0.5 | 300 | 1.84E-02 * | 0.0144 | 0.0173 | whole genome | 0.2 | 10 | 0.0225 | 0.0263 | 0.2267 | 0.0976 |
| NETRIN1 signaling | 0.5 | 500 | 1.93E-02 * | 0.0141 | 0.0170 | whole genome | 0.2 | 10 | 0.0225 | 0.0263 | 0.2269 | 0.0976 |
| NETRIN1 signaling | 0.5 | 1000 | 1.93E-02 * | 0.0141 | 0.0170 | whole genome | 0.2 | 10 | 0.0225 | 0.0263 | 0.2269 | 0.0976 |
| NETRIN1 signaling | 0.5 | 6000 | 1.91E-02 * | 0.0142 | 0.0171 | whole genome | 0.2 | 10 | 0.0225 | 0.0263 | 0.2268 | 0.0976 |
| NETRIN1 signaling | 1 | 10 | 5.70E-02 | 0.0076 | 0.0104 | whole genome | 0.2 | 10 | 0.0231 | 0.0267 | 0.2302 | 0.0979 |
| NETRIN1 signaling | 1 | 20 | 6.09E-02 | 0.0071 | 0.0097 | whole genome | 0.2 | 10 | 0.0232 | 0.0268 | 0.2293 | 0.0979 |
| NETRIN1 signaling | 1 | 50 | 4.39E-02 * | 0.0090 | 0.0121 | whole genome | 0.2 | 10 | 0.0230 | 0.0266 | 0.2276 | 0.0978 |
| NETRIN1 signaling | 1 | 200 | 4.85E-02 * | 0.0088 | 0.0119 | whole genome | 0.2 | 10 | 0.0230 | 0.0267 | 0.2281 | 0.0978 |
| NETRIN1 signaling | 1 | 300 | 4.94E-02 * | 0.0087 | 0.0119 | whole genome | 0.2 | 10 | 0.0230 | 0.0267 | 0.2282 | 0.0978 |
| NETRIN1 signaling | 1 | 500 | 4.84E-02 * | 0.0089 | 0.0120 | whole genome | 0.2 | 10 | 0.0230 | 0.0267 | 0.2281 | 0.0978 |
| NETRIN1 signaling | 1 | 1000 | 4.85E-02 * | 0.0088 | 0.0120 | whole genome | 0.2 | 10 | 0.0230 | 0.0267 | 0.2281 | 0.0978 |
| NETRIN1 signaling | 1 | 6000 | 4.85E-02 * | 0.0088 | 0.0119 | whole genome | 0.2 | 10 | 0.0230 | 0.0267 | 0.2281 | 0.0978 |

* significant results. Red highlighted line: largest variance explained by NETRIN1 PRS.

**Table S11. AUC statistics of PRSs.**

| **LD Clumping** | **Source of SNPs** | ***P* Cutoff** | **AUC** | **$N_{snps}$** |
|---|---|---|---|---|
| no | NETRIN1 signaling | 0.01 | 0.517 | 17 |
| no | NETRIN1 signaling | 0.05 | 0.522 | 104 |
| no | NETRIN1 signaling | 0.10 | 0.524 | 174 |
| no | NETRIN1 signaling | 0.20 | 0.530 | 328 |
| no | NETRIN1 signaling | 0.50 | 0.532 | 721 |
| no | NETRIN1 signaling | 1.00 | 0.529 | 1562 |
| yes | NETRIN1 signaling | 0.01 | 0.518 | 12 |
| yes | NETRIN1 signaling | 0.05 | 0.525 | 43 |
| yes | NETRIN1 signaling | 0.10 | 0.520 | 71 |
| yes | NETRIN1 signaling | 0.20 | 0.521 | 142 |
| yes | NETRIN1 signaling | 0.50 | 0.524 | 291 |
| yes | NETRIN1 signaling | 1.00 | 0.522 | 489 |
| no | whole genome | 0.01 | 0.508 | 5469 |
| no | whole genome | 0.05 | 0.511 | 24443 |
| no | whole genome | 0.10 | 0.515 | 47026 |
| no | whole genome | 0.20 | 0.519 | 90620 |
| no | whole genome | 0.50 | 0.520 | 215698 |
| no | whole genome | 1.00 | 0.521 | 419635 |
| yes | whole genome | 0.01 | 0.498 | 2819 |
| yes | whole genome | 0.05 | 0.516 | 11170 |
| yes | whole genome | 0.10 | 0.522 | 20212 |
| yes | whole genome | 0.20 | 0.527 | 35909 |
| yes | whole genome | 0.50 | 0.524 | 73298 |
| yes | whole genome | 1.00 | 0.527 | 110590 |

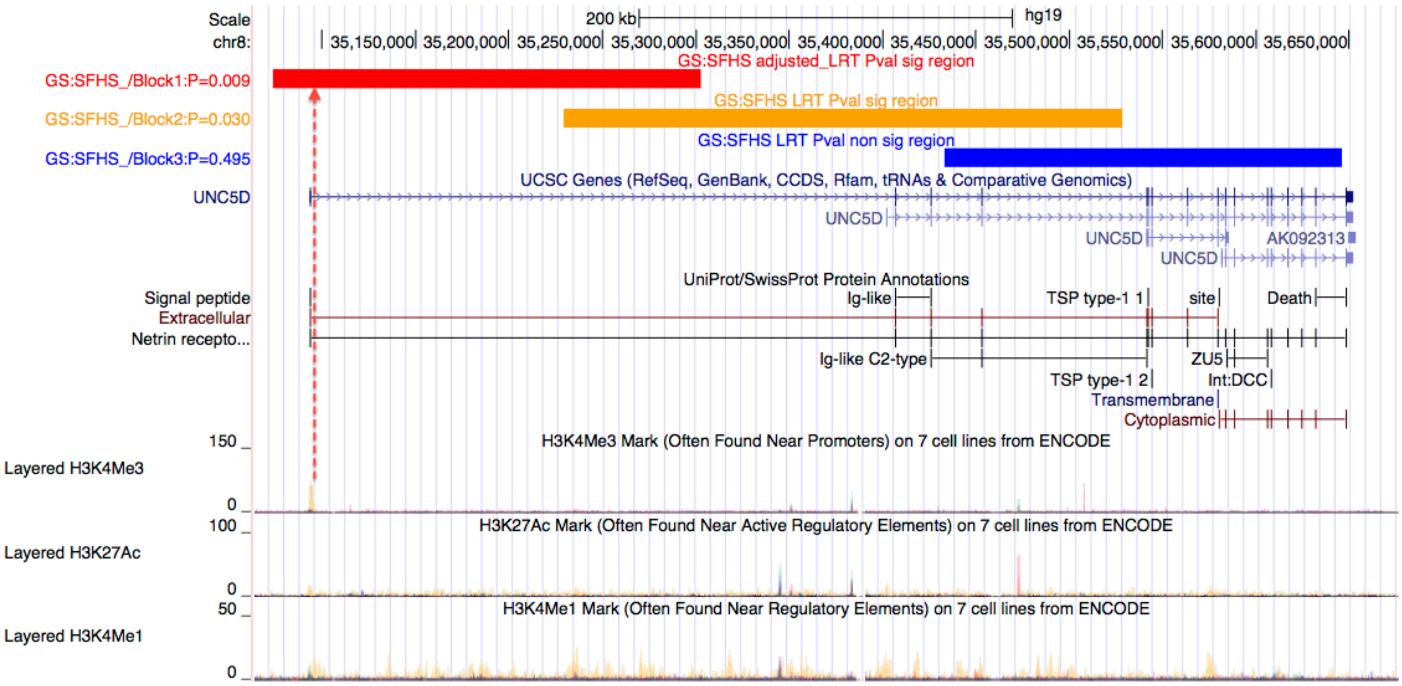


**Figure S1. Genic region in *UNC5D* showing blocks used in REACTA in GS:SFHS.** Three blocks were defined (window sizes = 200 SNPs, average block size = 247 kb). Blue bar: insignificant region in LRT. Orange bar: significant region in LRT. Red bar: significant region in LRT after Bonferroni correction. Red dotted line: significant Block 1 in *UNC5D* that overlaps with H3K4me3 signal region.

**Description of the Symbols Used in the Main Text:**

$\mathbf{N_{FDR}}$: number of the tests used in FDR correction

$\mathbf{N_{bonf}}$: number of the tests used in Bonferroni correction

$\boldsymbol{P_{path}}$ **:** *P* value of the pathway analysis

$\boldsymbol{P_{path\_bonf}}$ **:** adjusted *P* value (Bonferroni method) of the pathway analysis

$\boldsymbol{P_{lrt}}$: nominal *P* value from the log-likelihood ratio test

$\boldsymbol{P_{lrt\_FDR}}$: adjusted *P* value (FDR method) of the log-likelihood ratio test

$\boldsymbol{P_{lrt\_bonf}}$ : adjusted *P* value (Bonferroni method) of the log-likelihood ratio test

$\boldsymbol{P_{perm}}$**:** *P* value of the permutation analysis

## Supplemental References


1. First MB, Spitzer RL, Gibbon M, Williams JB (2002) Structured clinical interview for DSM-IV-TR axis I disorders, research version, patient edition. SCID-I/P.
2. Major Depressive Disorder Working Group of the Psychiatric GC, Ripke S, Wray NR, Lewis CM, Hamilton SP, Weissman MM, *et al.* (2013) A mega-analysis of genome-wide association studies for major depressive disorder. Mol Psychiatry 18: 497-511.
3. Milaneschi Y, Lamers F, Peyrot WJ, Abdellaoui A, Willemsen G, Hottenga JJ, *et al.* (2015) Polygenic dissection of major depression clinical heterogeneity. Mol Psychiatry.
4. Howie B, Marchini J, Stephens M (2011) Genotype Imputation with Thousands of Genomes. G3-Genes Genomes Genetics 1: 457-469.
5. Howie BN, Donnelly P, Marchini J (2009) A Flexible and Accurate Genotype Imputation Method for the Next Generation of Genome-Wide Association Studies. Plos Genetics 5.
6. Delaneau O, Marchini J, Zagury JF (2012) A linear complexity phasing method for thousands of genomes. Nature Methods 9: 179-181.
7. Juraeva D, Haenisch B, Zapatka M, Frank J, Investigators G, group P-GSw, *et al.* (2014) Integrated Pathway-Based Approach Identifies Association between Genomic Regions at CTCF and CACNB2 and Schizophrenia. PLoS Genet 10: e1004345.
8. Veyrieras JB, Kudaravalli S, Kim SY, Dermitzakis ET, Gilad Y, Stephens M, *et al.* (2008) High-resolution mapping of expression-QTLs yields insight into human gene regulation. PLoS Genet 4: e1000214.
9. Subramanian A, Tamayo P, Mootha VK, Mukherjee S, Ebert BL, Gillette MA, *et al.* (2005) Gene set enrichment analysis: a knowledge-based approach for interpreting genome-wide expression profiles. Proc Natl Acad Sci U S A 102: 15545-15550.
10. Elbers CC, van Eijk KR, Franke L, Mulder F, van der Schouw YT, Wijmenga C, *et al.* (2009) Using Genome-Wide Pathway Analysis to Unravel the Etiology of Complex Diseases. Genetic Epidemiology 33: 419-431.
11. Wang K, Li M, Hakonarson H (2010) ANNOVAR: functional annotation of genetic variants from high-throughput sequencing data. Nucleic Acids Res 38: e164.
12. Chen LS, Hutter CM, Potter JD, Liu Y, Prentice RL, Peters U, *et al.* (2010) Insights into Colon Cancer Etiology via a Regularized Approach to Gene Set Analysis of GWAS Data. American Journal of Human Genetics 86: 860-871.
13. Segre AV, Consortium D, investigators M, Groop L, Mootha VK, Daly MJ, *et al.* (2010) Common inherited variation in mitochondrial genes is not enriched for associations with type 2 diabetes or related glycemic traits. PLoS Genet 6.
14. Cabrera CP, Navarro P, Huffman JE, Wright AF, Hayward C, Campbell H, *et al.* (2012) Uncovering networks from genome-wide association studies via circular genomic permutation. G3 (Bethesda) 2: 1067-1075.
15. Nagamine Y, Pong-Wong R, Navarro P, Vitart V, Hayward C, Rudan I, *et al.* (2012) Localising loci underlying complex trait variation using Regional Genomic Relationship Mapping. PLoS One 7: e46501.
16. Karolchik D, Barber GP, Casper J, Clawson H, Cline MS, Diekhans M, *et al.* (2014) The UCSC Genome Browser database: 2014 update. Nucleic Acids Res 42: D764-770.
17. Dudbridge F (2013) Power and predictive accuracy of polygenic risk scores. PLoS Genet 9: e1003348.
18. Euesden J, Lewis CM, O'Reilly PF (2015) PRSice: Polygenic Risk Score software. Bioinformatics 31: 1466-1468.
19. Purcell S, Neale B, Todd-Brown K, Thomas L, Ferreira MAR, Bender D, *et al.* (2007) PLINK: A tool set for whole-genome association and population-based linkage analyses. American Journal of Human Genetics 81: 559-575.
